# Gating and modulation of an inward-rectifier potassium channel

**DOI:** 10.1085/jgp.202213085

**Published:** 2022-12-16

**Authors:** Vishwanath Jogini, Morten Ø. Jensen, David E. Shaw

**Affiliations:** 1 D. E. Shaw Research, New York, NY, USA; 2 Department of Biochemistry and Molecular Biophysics, Columbia University, New York, NY, USA

## Abstract

Inward-rectifier potassium channels (Kirs) are lipid-gated ion channels that differ from other K^+^ channels in that they allow K^+^ ions to flow more easily into, rather than out of, the cell. Inward rectification is known to result from endogenous magnesium ions or polyamines (e.g., spermine) binding to Kirs, resulting in a block of outward potassium currents, but questions remain regarding the structural and dynamic basis of the rectification process and lipid-dependent channel activation. Here, we present the results of long-timescale molecular dynamics simulations starting from a crystal structure of phosphatidylinositol 4,5-bisphosphate (PIP_2_)-bound chicken Kir2.2 with a non-conducting pore. After introducing a mutation (G178R) that is known to increase the open probability of a homologous channel, we were able to observe transitions to a stably open, ion-conducting pore, during which key conformational changes occurred in the main activation gate and the cytoplasmic domain. PIP_2_ binding appeared to increase stability of the pore in its open and conducting state, as PIP_2_ removal resulted in pore closure, with a median closure time about half of that with PIP_2_ present. To investigate structural details of inward rectification, we simulated spermine binding to and unbinding from the open pore conformation at positive and negative voltages, respectively, and identified a spermine-binding site located near a previously hypothesized site between the pore cavity and the selectivity filter. We also studied the effects of long-range electrostatics on conduction and spermine binding by mutating charged residues in the cytoplasmic domain and found that a finely tuned charge density, arising from basic and acidic residues within the cytoplasmic domain, modulated conduction and rectification.

## Teaser

Based on the results of long-timescale molecular dynamics simulations, Jogini et al. provide atomic-level mechanistic and structural descriptions of lipid-dependent gating and spermine-induced inward rectification of the inward-rectifier potassium channel Kir2.2.

## Introduction

**Figure 1.**
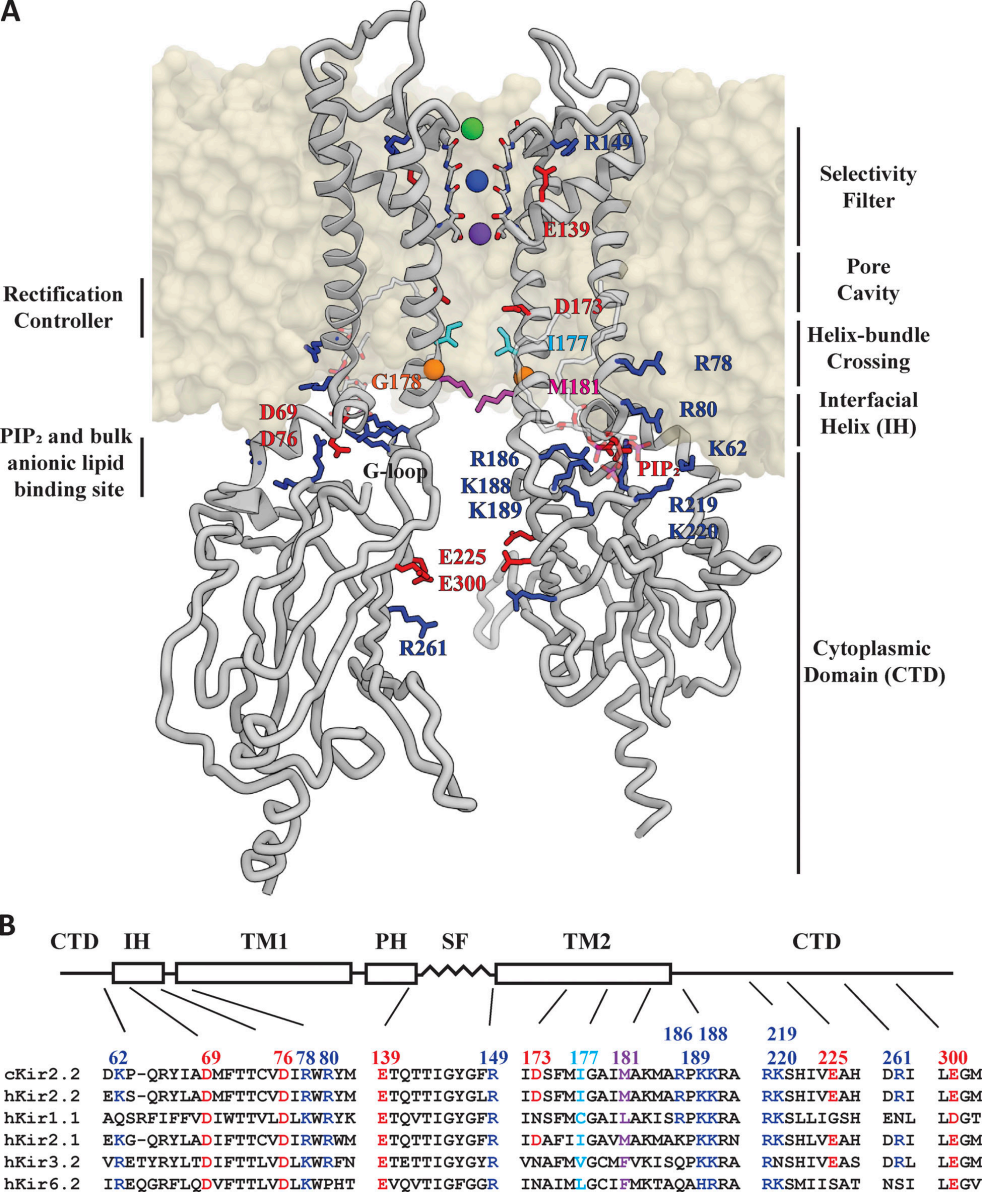
**Important structural features of Kir channels. (A)** Diagonally opposite subunits of membrane-embedded cKir2.2 (taken from Sim. 2; t = 0). Side chains that are in the helix-bundle crossing (activation gate), involved in rectification, or are part of the lipid-binding sites, are represented as sticks. PIP_2_ molecules bound to the channel are represented as semi-transparent sticks. C_α_ atoms of the mutated G178 residue are shown as orange spheres. SF residues 144–148 are represented as sticks, with SF-bound ions individually colored. E139 and R149, which are located behind the SF and highly conserved across the Kir family, are represented as red and blue sticks, respectively. **(B)** Sequence alignment of key functional regions in Kir channels that are strongly rectifying (cKir2.2, hKir2.2, and hKir2.1) or weakly rectifying (hKir1.1, hKir3.2, and hKir6.2), gated by PIP2 (cKir2.2, hKir2.2, and hKir2.1), ATP (hKir6.2), pH (hKir1.1), or G-protein (hKir3.2). Red, cyan, and blue highlighted residues are all known to be crucial for gating and/or rectification.

Inward-rectifier potassium channels (Kirs) are lipid-gated ion channels that, in contrast with most K^+^ channels, allow K^+^ ions to flow more easily into the cell than out of the cell. They are present in both excitable and non-excitable membranes, and assume diverse physiological roles depending on the channel subtype and location. In cardiac myocytes, for example, they prolong action potentials, in pancreatic β cells they control insulin release, and in kidney epithelial cells they regulate salt/water uptake (Hibino et al., 2010). Kirs function as tetramers, with each subunit containing (1) a transmembrane (TM) pore domain composed of two TM helices connected by a pore loop that contains the selectivity filter (SF; having a sequence identical to that of other K^+^-selective channels), and (2) large N- and C-terminal regions that together constitute the cytoplasmic domain (CTD), which includes a CTD pore (Tao et al., 2009; Hansen et al., 2011; Fig. 1 provides an overview of the relevant domains, binding sites, and residues of Kir channels that are discussed throughout this article). Mutations in Kirs are associated with various channelopathies; for instance, certain mutations in the ATP-dependent Kir channel Kir6.2 are known to be a cause of neonatal diabetes (Hattersley and Ashcroft, 2005), and mutations in the outer medullary potassium channel Kir1.1 (ROMK) lead to a type of renal failure known as Bartter syndrome (Derst et al., 1997).

All Kirs require phosphatidylinositol 4,5-bisphosphate (PIP_2_) to function (Hibino et al., 2010; Huang et al., 1998; Zhang et al., 1999), and different Kir isoforms are additionally modulated physiologically by other negatively charged lipids (Lee et al., 2013; Choe et al., 2001), ATP (Terzic et al., 1995), pH (Fakler et al., 1996; Choe et al., 1997), or G-proteins (He et al., 1999; Albsoul-Younes et al., 2001). PIP_2_-dependence of Kir2.2 (as with all Kirs) is mediated by conserved arginine (R)- and lysine (K)-rich membrane-exposed regions, all located on the intracellular side of the channel, that allow these channels to sense and recruit PIP_2_. ATP, pH, and G-protein sensors, which are present in certain Kirs (but not Kir2.2), are also located intracellularly, with some residing within the CTD (Hibino et al., 2010). In Kir2.x channels, PIP_2_ is essential for channel activation (i.e., for pore opening; Cheng et al., 2011), whereas channel open probabilities and ion permeation rates are modulated by other regulatory, negatively charged phospholipids and by the permeating ions themselves (Lee et al., 2013; Choe et al., 2001; Lee et al., 2016).

X-ray crystallography has revealed the architecture of homo-tetrameric, PIP_2_-bound chicken Kir2.2 (cKir2.2; Hansen et al., 2011) and mouse G-protein–activated Kir3.2 (Whorton and MacKinnon, 2011). Although these structures were obtained under activating conditions (i.e., in the presence of PIP_2_), they both capture the channel with its pore in a non-conducting conformation, with four agonizing PIP_2_ molecules bound near the membrane-facing R/K-rich regions (one per subunit). A PIP_2_-depleted (apo) cKir2.2 structure has also been resolved (Tao et al., 2009), and the CTD is closer to the TM pore domain in the PIP_2_-bound cKir2.2 structure than in the apo structure. Pore opening with PIP_2_ bound has thus been proposed to involve movement of the CTD toward the TM pore (Hansen et al., 2011). Exact details about where negatively charged lipid molecules bind and how they may promote channel activation and pore opening, however, remain partly unknown.

In all Kir channels, magnesium ions and endogenous polyamines, such as spermine (SPM) and spermidine, block the ion-conducting pore at depolarizing voltages exceeding the potassium ion reversal potential, resulting in the cessation of ion conduction (Lopatin et al., 1994). At negative voltages, however, these blockers dislodge from the pore, allowing for inward conduction. This voltage-dependent block forms the basis of inward rectification in Kirs (Lopatin et al., 1994; Ficker et al., 1994; Taglialatela et al., 1995), yet the exact structural details of this block, and relief from it, have been unknown. In addition, possible locations of polyamine binding have been proposed to be either below the TM pore cavity and near the CTD (Guo and Lu, 2003), or between the (central) pore cavity and the SF region (Kurata et al., 2013), with the exact details remaining largely unresolved (Nichols and Lee, 2018; Baronas and Kurata, 2014; Chen et al., 2020). One study, for example, identified a negatively charged aspartate residue in Kir2.1 located in the TM pore cavity (corresponding to D173 in cKir2.2) that appears to contribute to time-dependent polyamine binding (Wible et al., 1994); when this residue was charge-neutralized, only a partial polyamine block remained. That this partial block remained suggests that charged residues in the CTD may also contribute to polyamine binding and partial channel block in Kir2.2. In ROMK, which lacks a D173-corresponding acidic TM–pore-cavity residue, only shallow inward rectification occurs, and it is only upon introduction of such a rectification-controlling residue that strong inward rectification was observed (Guo and Lu, 2003; Wible et al., 1994; Pegan et al., 2005; Fujiwara and Kubo, 2006).

Functional studies have suggested that certain charged residues in the CTD also modulate ion permeation (Pegan et al., 2005; Fujiwara and Kubo, 2006), a finding consistent with structural observations of a single file of potassium ions spanning the entire permeation pathway—the extended pore, which is composed of the CTD pore and the TM pore—and interacting with these residues (Tao et al., 2009; Nishida et al., 2007). Moreover, ion and polyamine binding within the CTD appear to influence voltage-dependent permeation and polyamine block in the TM pore (Shin et al., 2005; Robertson et al., 2008).

Here, we report the use of long-timescale molecular dynamics (MD) simulations to investigate channel opening of cKir2.2. We observed key conformational changes—including slight rotation of the inner TM helix, splaying of both TM helices, and movement of the I177 and M181 activation gate residues—and ultimately obtained a PIP_2_-bound, putatively fully activated channel that exhibited steady-state ion conduction across an open pore. In our simulations of the activated channel in a PIP_2_-depleted system, the open pore transitioned into a closed, non-conducting conformation, indicating that PIP_2_ is indispensable for channel function (and in agreement with functional [Huang et al., 1998; Zhang et al., 1999] and structural [Tao et al., 2009; Hansen et al., 2011; Whorton and MacKinnon, 2011] studies). We also simulated the binding of SPM to the open, conducting pore of the fully activated channel, and observed cessation of ion conduction upon SPM binding. These simulations identified a SPM-binding site that is consistent with the approximate location of one of two previously hypothesized polyamine-binding sites—a finding that could potentially be helpful in resolving a lack of consensus in the literature (Guo and Lu, 2003; Kurata et al., 2013; Nichols and Lee, 2018; Baronas and Kurata, 2014). We then examined the voltage dependence of SPM binding and unbinding, providing structural and dynamic details of inward rectification. Results from additional simulations of cKir2.2 mutants with altered CTD residues suggest how certain charged residues in the CTD may modulate ion conductance and channel rectification.

## Materials and methods

### Simulations

As a starting point for our simulations, we used the 3.3-Å resolution structure of cKir2.2 in which the channel is in complex with one PIP_2_ molecule per subunit (PDB ID 3SPI; Hansen et al., 2011). Missing loops were built using Molecular Operating Environment (MOE; Vilar et al., 2008). The channel was embedded in a hydrated 1-palmitoyl-2-oleoyl-sn-glycero-3-phospho-choline (POPC) lipid bilayer, and its net charge was neutralized with overall ionic (KCl) concentrations of 0.2 M or 0.5 M. The four PIP_2_ molecules present in the crystal structure (one per subunit) were retained and 40 1-palmitoyl-2-oleoyl-sn-glycero-3-phospho-L-serine (POPS) molecules (amounting to ∼14% of the total bilayer composition) were added at random positions to the intracellular membrane leaflet to mimic the negatively charged phospholipid molecules that are known to enhance the channel open probability of the closely related hKir2.1 channel (Cheng et al., 2011). In two diagonally opposed subunits, the negatively charged residue D173, located in the pore cavity, was protonated to reduce the large negative charge density within the cavity region (whereas the two other D173 residues were left deprotonated); when all four D173 residues were left deprotonated, potassium ions were, at a lower applied voltage (*V* = 215 mV), typically trapped at this position, leading to transient, unphysiological pore block. This suggests that two D173 residues should be protonated. Protonation states of all titratable residues were otherwise chosen such that they corresponded to a pH value of 7.

The system contained ∼185,000 atoms, with initial measurements of approximately 108 × 108 × 140 Å^3^. To impose a transmembrane voltage difference, *V*, we applied constant electric fields of −0.05 ≤ *E* < 0.06 kcal mol^−1^ Å^−1^ e^−1^, corresponding to −310 ≤ *V* < 375 mV (Roux, 2008; Jensen et al., 2013; Gumbart et al., 2012). All simulations were performed on the special-purpose supercomputer Anton (Shaw et al., 2014), in the NPT ensemble at 310 K with 1 bar pressure. Temperature and pressure were controlled using the Martyna–Tobias–Klein (MTK) algorithm (Martyna et al., 1996) and the Nosé–Hoover thermostat (Hoover, 1985), respectively, applied using the multigrator framework (Lippert et al., 2013). Water molecules and all bond lengths to hydrogen atoms were constrained using an implementation (Lippert et al., 2007) of M-SHAKE (Kräutler et al., 2001). Van der Waals and short-range electrostatic interactions were cut off at 9 and 13 Å, respectively. Long-range electrostatic interactions were calculated using the *u*-series method (Predescu et al., 2020) with a 32 × 32 × 64 FFT mesh. The simulation time step was 2.5 fs, and long-range electrostatics were evaluated every third time step. The aggregate simulation time was 5.1 ms, with individual simulation times of ∼2–200 µs. Additional simulation details are provided in Table S1.

### Force fields

The CHARMM22 force field (MacKerell et al., 1998) was used for the protein and ions, with the CMAP correction for the protein backbone (MacKerell et al., 2004), and with modified partial charges on Glu, Asp, and Arg residues (Jensen et al., 2012). We used the TIP3P model for water (Jorgensen et al., 1983), and the CHARMM36 parameter set for all lipid molecules, including PIP_2_ and POPS (Klauda et al., 2010). We used the CHARMM General Force Field (Vanommeslaeghe et al., 2010; Yu et al., 2012) for SPM, for which all parameters are listed in Table S2.

As in our previous long-timescale simulations of other potassium channels (Jensen et al., 2013), we observed here that the SF was unstable in our cKir2.2 simulations; notably, the Y146 carbonyl group underwent unphysical backbone rotations when timescales extended beyond a few microseconds, causing the SF to collapse. Similar instability of the cKir2.2 SF has been observed in simulations performed by others, although on shorter timescales (Zangerl-Plessl et al., 2020). In order to ensure that the SF remained structurally stable during our long-timescale simulations, backbone torsional (ϕ, ψ) corrections were applied to SF residues 143–147 to lower the backbone torsional potential by 4.6 kcal mol^−1^ at the crystallographic ϕ and ψ minima relative to the value of this potential at θ + 180° (Jensen et al., 2013; Song et al., 2018). In a subset of simulations, only ϕ torsional corrections were applied, and only to two SF residues—G145 and G147 (see Table S1). Aside from these torsional corrections, no modifications were made to the force fields used in our simulations.

### Online supplemental material

**Figure S1.**
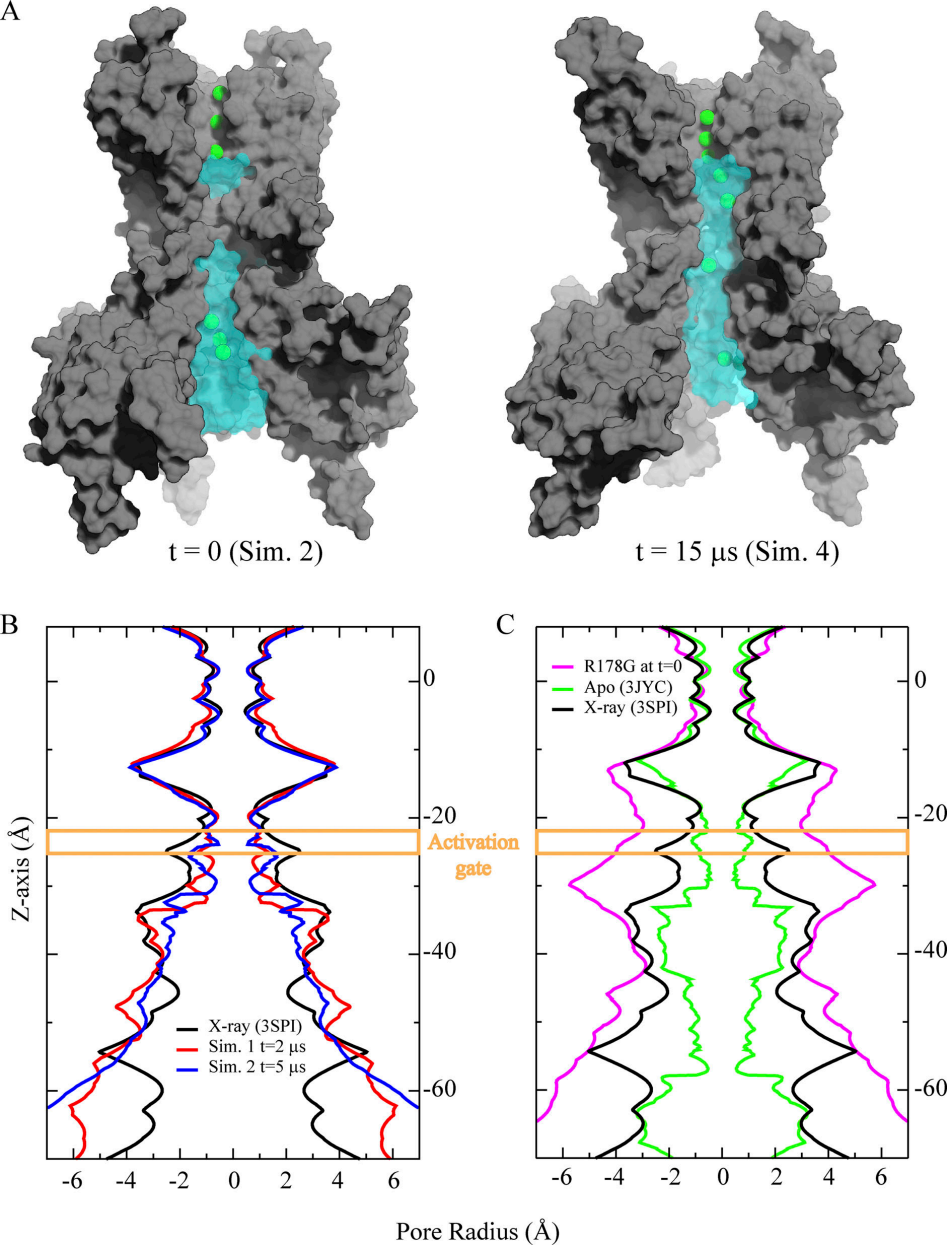
**Pore radius profiles. (A)** Pore hydration (transparent cyan color) in the closed and open pore conformations; only three protein subunits are shown (grey surface), and permeating potassium ions appear as green spheres. **(B)** Pore radius profiles of the PIP_2_-agonized crystal structure (PDB ID 3SPI), from a snapshot 2 µs into Sim. 1, and from a snapshot 5 µs into Sim. 2. Together, these profiles suggest a transition from the open, non-conducting conformation to a preactivated, semi-open pore conformation, and subsequently to a fully closed pore conformation. **(C)** Pore radius profile of the open-pore conformation obtained in simulations of the R178G activation gate mutant (Sim. 4) compared with the crystal structures in the presence (PDB ID 3SPI) and absence (PDB ID 3JYC) of PIP_2_, with preactivated, semi-open, and closed pore conformations. The pore radius profiles were generated using HOLE (Smart et al., 1996).

**Figure S2.**
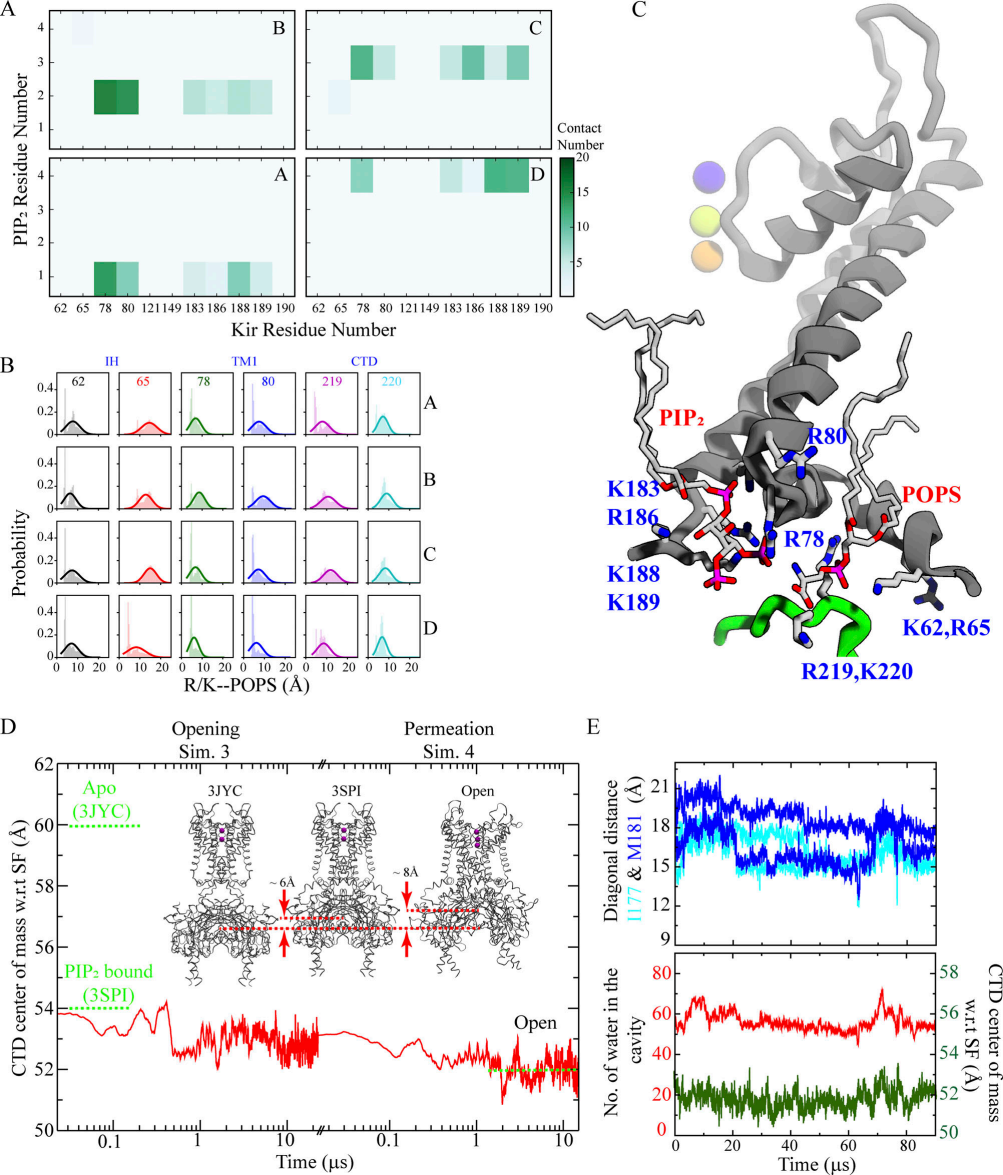
**Lipid interactions with the R/K-rich region, movement of the CTD, and open state stability. (A)** Interactions between negatively charged PIP_2_ and positively charged R/K residues are preserved during channel activation and pore opening (Sim. 3); PIP_2_ atoms within 4 Å of an R/K residue were defined as interacting. **(B)** Per-subunit (i.e., A–D) distribution of POPS:P distances to K62:Cζ/R65:Nζ (IH), R78:Nζ/R80:Nζ (TM1), and R219:Nζ/K220:Cζ (CTD). **(C)** Snapshot of one TM subunit (gray) and part of the CTD of an adjacent subunit (green) highlighting PIP_2_ and POPS-interacting (R/K) residues. SF-bound ions are shown as colored spheres. **(D)** CTD-SF center of mass separation during pore opening in simulations of the activation gate G178R mutant (Sim. 3) and upon back mutation of this mutant to the WT (Sim. 4). Green dotted lines are reference distances from the apo (PDB ID 3JYC) and PIP_2_-bound (PDB ID 3SPI) crystal structures (Tao et al., 2009; Hansen et al., 2011). Inset: Two crystal structures and a simulation snapshot of the open state obtained in Sim. 4; dotted red line indicates the CTD movement toward the TM domains (all structures were TM-aligned). **(E)** Stability of the open pore conformation (Sim. 4) assessed by measuring the C_α_–C_α_ diagonal distance at activation gate residues I177 and M181 (top), the pore cavity hydration, and the CTD-SF center of mass separation (bottom).

**Figure S3.**
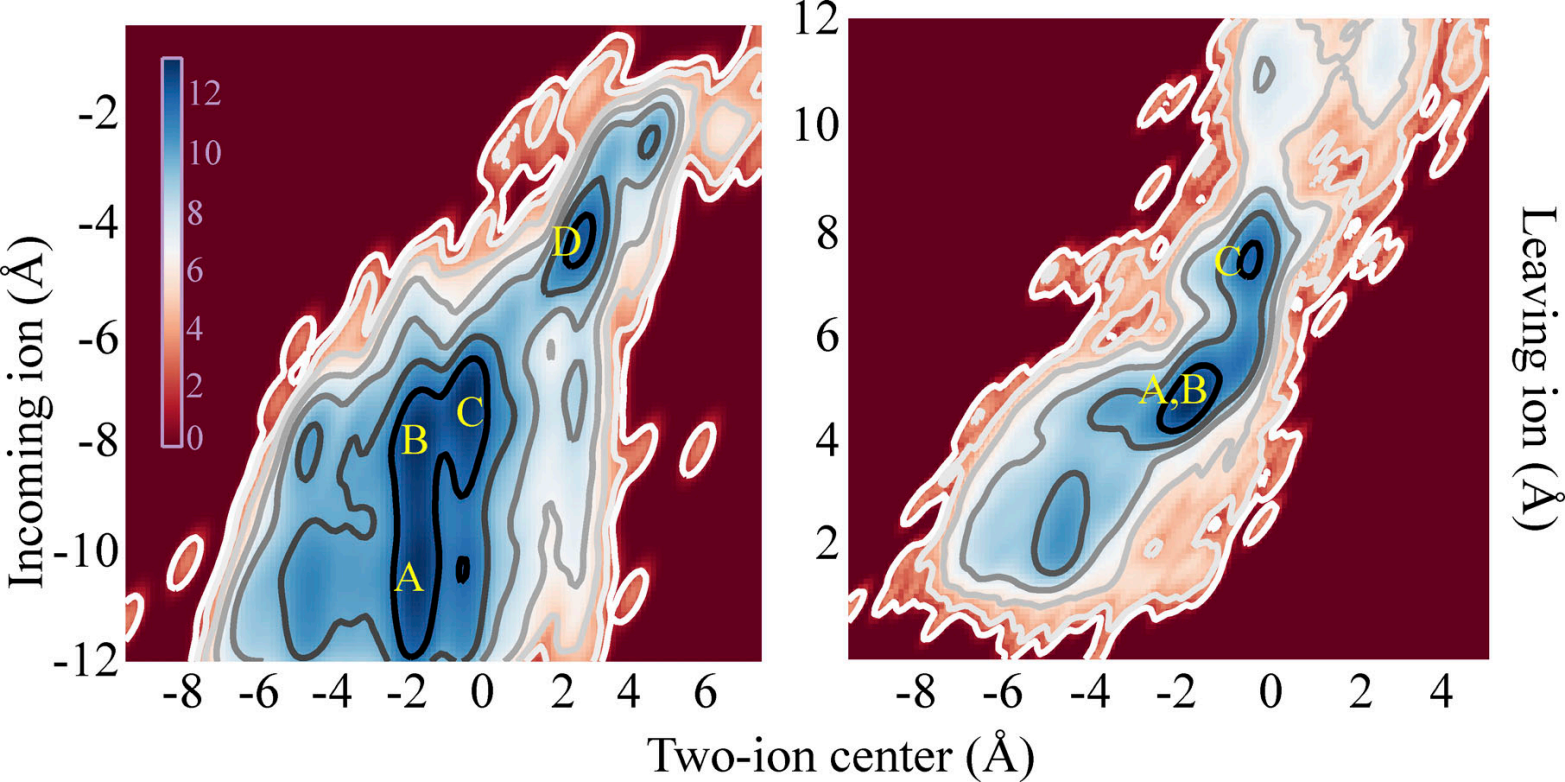
**Permeation mechanism in the E225A/E300A double mutant.** Position of the incoming ion vs. the centroid of the two SF-bound ions located above it (left), and position of the leaving ion vs. the centroid of the two SF-bound ions located below it (right). Color bar is in units of log(ρ[x,y]); ρ[x,y] is the two-dimensional histogram of ion positions averaged over the entire simulation time of Sim. 26 and Sim. 27. Blue minima represent the four predominant three-ion and four-ion configurations in the knock-on permeation mechanism. All observed configurations are similar to those observed during permeation across the WT (see Fig. 1 E), indicating that the predominant permeation mechanism in this mutant is unchanged from that observed in the WT.

**Figure S4.**
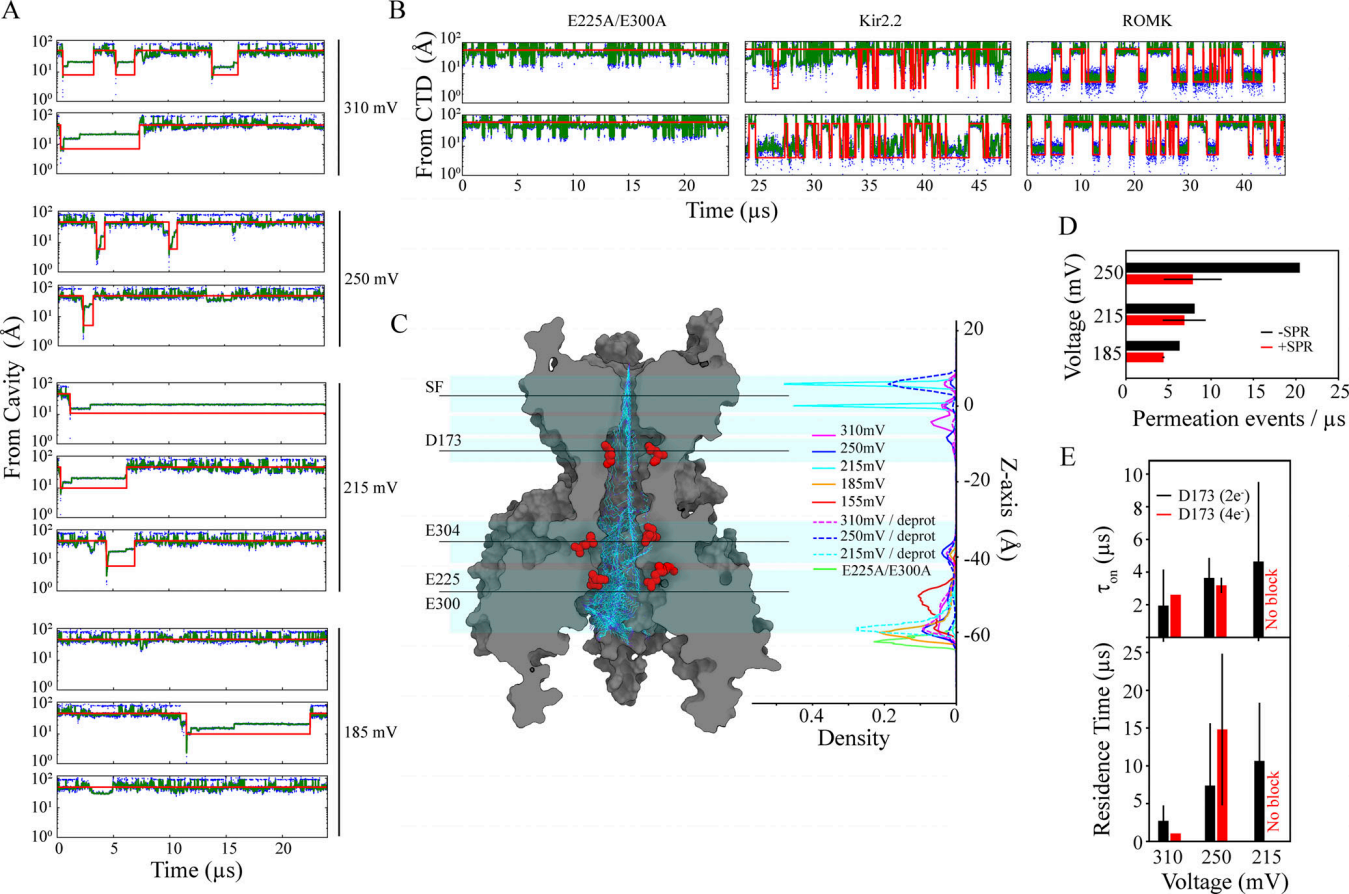
**SPM-binding kinetics. (A)** Voltage-dependent SPM binding times (Sims. 31, 32, 35, 36, 39–41, and 43–45), with binding defined as when the distance from SPM to the D173 center of mass (C_α_ atoms) was 10 Å or less (30-ns running medians). **(B)** SPM binding (at 310 mV) to the CTD in the E225A/E300A CTD double mutant (Sims. 91 and 92), in WT Kir2.2 (Sims. 31 and 32), and in ROMK (Sims. 153 and 154). Binding was defined as when the distance from SPM to the E225/E300 center of mass (C_α_ atoms) was 10 Å or less (30-ns running medians). **(C)** SPM snapshots (cyan) during binding (Sim. 35) to the extended pore spanning the entire protein (gray cut-through surface). SPM center of mass number density along the (extended) pore-axis in WT with two or four rectification controller (D173) residues deprotonated, and in the E225A/E300A CTD double mutant. **(D)** Ion permeation events per unit time in the presence and absence of SPM (Sims. 36–46, red; Sims. 9–11, black) at different depolarizing voltages. **(E)** Voltage-dependent SPM binding (Fig. S4 A) and SPM residence times at the pore cavity-SF region with two and with four D173 residues deprotonated (Sims. 31–42, 150–152).

**Figure S5.**
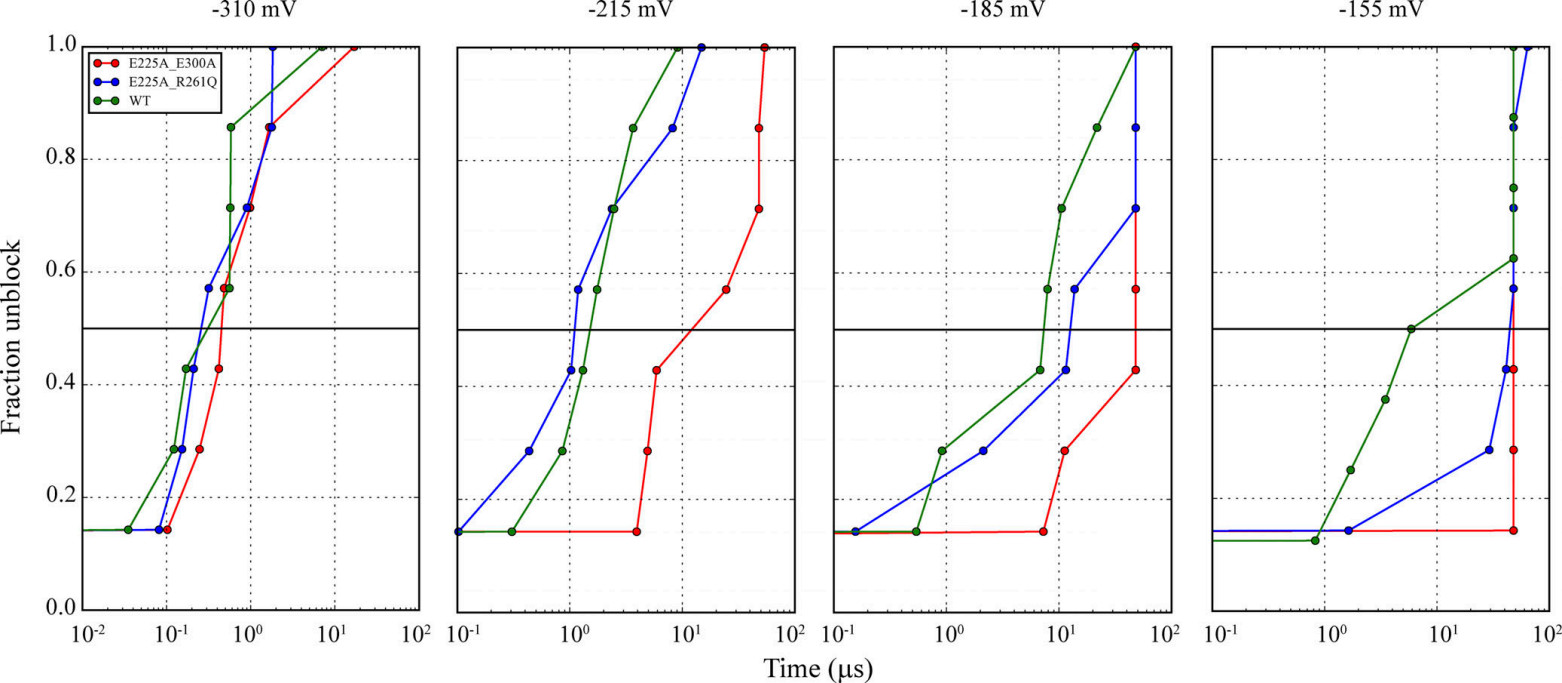
**Impact of the CTD charge density on SPM unbinding.** Fraction of simulations relieved from SPM pore block at different voltages observed in simulations of the WT (Sims. 61–81, 83–90) and in the E225A/E300A and E225A/R261Q CTD double mutants (Sims. 93–120 and Sims. 121–148, respectively). SPM unbinding times were identified when the distance from SPM to the D173 center of mass (C_α_ atoms) exceeded 20 Å (30 ns-running medians).

**Figure S6.**
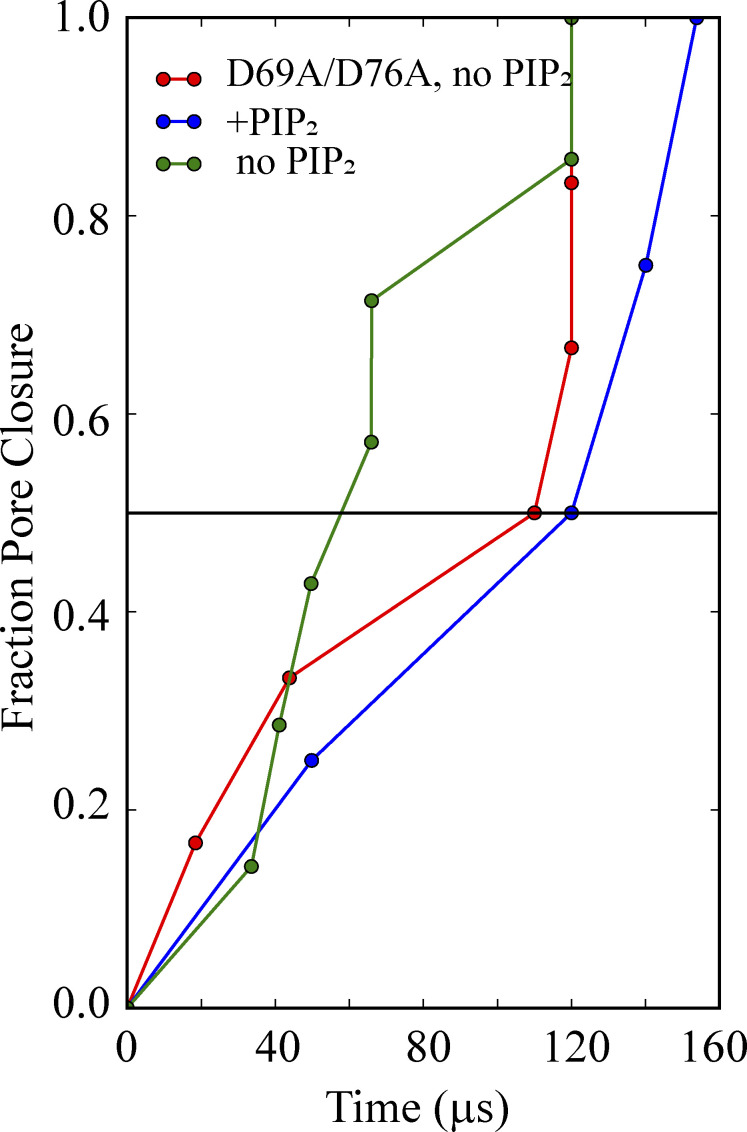
**IH residues D69 and D76 stabilize the closed pore conformation in the deactivated state.** Fraction of WT simulations in which pore closure was observed, performed in the presence and absence of PIP_2_ (Sims. 4–6, Sim. 11, and Sims. 13–19, respectively), and in simulations of the D69A/D76A IH double mutant performed without PIP_2_ (Sims. 20–25). The instance of pore closure was defined as the simulation time when the number of water molecules in the pore cavity decreased to 35 or less (the average hydration of a stably open pore conformation was 56 ± 8).

Fig. S1 quantifies the extent of pore opening in Kir2.2 by (1) comparing pore hydration in closed and open pore conformations, obtained from the simulations, with the PIP_2_-bound, semi-open x-ray structure (PDB ID 3SPI); and (2) comparing pore radius profiles of PIP2-bound and PIP2-depleted crystal structures (PDB IDs 3SPI and 3JYC, respectively; Tao et al., 2009; Hansen et al., 2011) with a simulation snapshot of an open channel (obtained by introducing the G178R activation gate mutation, simulating opening, and then back-mutating to wild type). Fig. S2 summarizes the lipid (i.e., PIP_2_ and POPS) interactions with the Kir2.2 channel and quantifies both the movement of the CTD during channel activation and the stability of the activated channel. Fig. S3 shows diagrammatically the ion permeation mechanism in the CTD E225A/E300A double mutant (the mechanism is the same as for the WT channel). Figs. S4 and S5 summarize the kinetics of voltage-dependent SPM binding and unbinding and how it is modulated by the charge density on the CTD. Fig. S6 summarizes the role of two aspartate residues (D69 and D76) located on the interfacial helix (IH) in channel activation and deactivation. Video 1 shows conformational changes during channel activation (pore opening) in the G178R activation gate mutant. Video 2 shows ion permeation (under depolarizing conditions) across the SF of the activated WT channel (with an open pore). Videos 3 and 4 show channel deactivation in the absence of PIP_2_, highlighting activation gate and IH movements. Videos 5 and 6 show SPM binding and unbinding under depolarizing and hyperpolarizing conditions. Table S1 includes key details about the simulated systems and the primary outcome of each simulation presented in this study. SPM force field parameters are listed in Table S2.

## Results

### Pore opening and ion permeation

**Figure 2.**
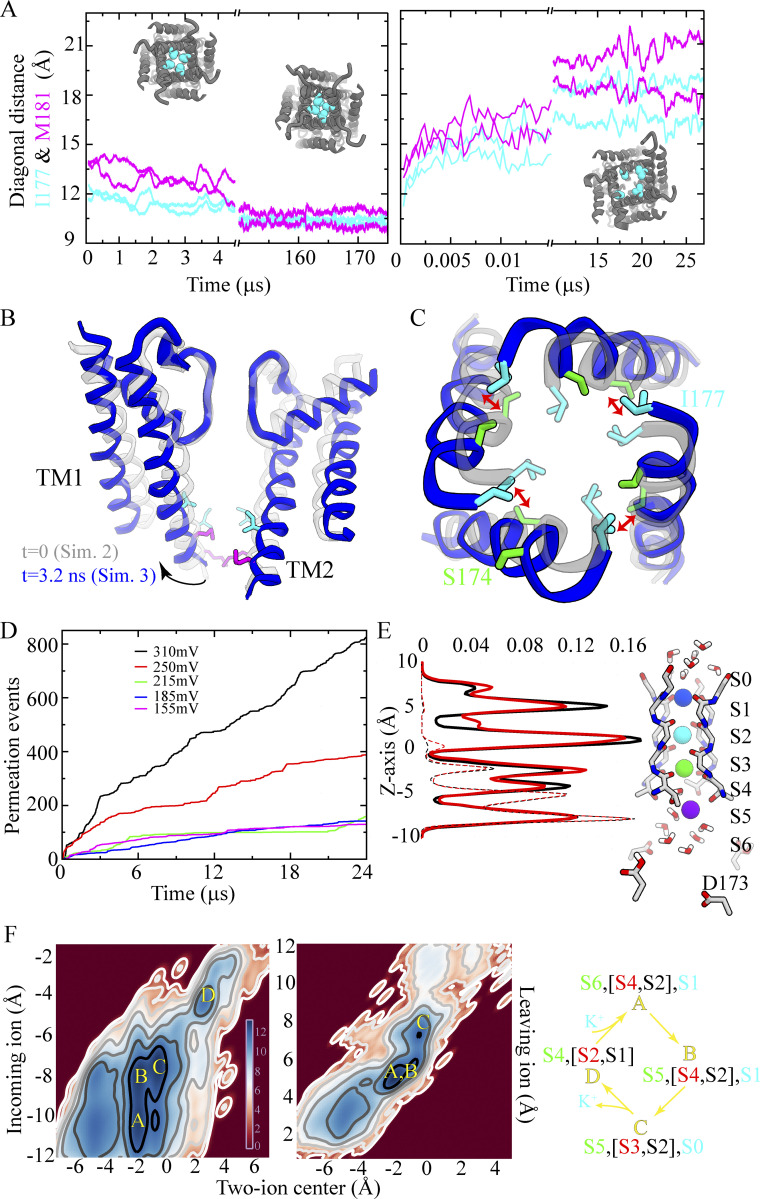
**Activation gate opening and permeation. (A)** Activation gate closure (WT; Sim. 2) and opening (G178R; Sim. 3) measured by the C_α_–C_α_ diagonal distance at gate residues I177 and M181. Insets: Intracellular view of the TM pore with I177 residues indicated as spheres and the TM region in grey. **(B)** Movement of the inner TM helices illustrating the main activation gate conformational change with only two diagonally opposed subunits shown. **(C)** Pore opening viewed from the intracellular side with the same representations as in B; double-headed arrow indicates S174–I177 positional interchange. **(D)** Ion permeation events obtained under depolarizing voltages of different magnitude (Sims. 8–12). **(E)** Density profiles of ion (solid lines) and water (dashed lines) permeating across the SF (Sim. 7, black; Sim. 8, red; both at 310 mV) with a simulation snapshot of the ion-occupied SF (residues 144–148) and the rectification controller D173, located in the pore cavity, with two of the four D173 residues (de)protonated; ions are individually colored, and water molecules appear in red and white. **(F)** Position of the incoming ion vs. the centroid of the two SF-bound ions located above it (left), and position of the leaving ion vs. the centroid of the two SF-bound ions located below it (right). Color bar in units of log(ρ[*x*,*y*]); ρ[*x*,*y*] is the two-dimensional histogram of ion positions averaged over the entire simulation time of Sim. 7 and Sim. 8. Blue minima represent the four predominant three-ion and four-ion configurations observed during permeation that occurs by a knock-on mechanism. S0–S6 denote SF ion-binding sites, and the predominant permeation mechanism is summarized in the right panel. Formation of the knock-on intermediate S5[S3,S2],S0 is the rate limiting step of the permeation.

Initially, we performed long-timescale MD simulations starting from a PIP_2_-bound cKir2.2 structure (Hansen et al., 2011) in which the pore appears to be non-conducting (Sims. 1 and 2). POPS lipid molecules were included in Sim. 2, as they are known to increase channel open probabilities and single-channel conductance of inward rectifier Kir channels (Lee et al., 2013; Choe et al., 2001; Cheng et al., 2011; Lee et al., 2016). In both of these simulations, rapid pore closure occurred within a few microseconds of simulated time (Fig. 2 A and Table S1). The small pore opening in the PIP_2_-bound structure allowed for only a modest pore-cavity hydration in these two simulations, which appears to be consistent with experimental observations of relatively small currents with only PIP_2_ present (the additional presence of anionic bulk lipids in these experiments increased the currents, indicative of a relatively wider pore; Cheng et al., 2011). The degree of hydration was insufficient to withstand the attractive hydrophobic interaction between activation gate residues I177 and M181, resulting instead in a hydrophobic collapse of the activation gate and, consequently, complete pore closure (Fig. 2 A and Fig. S1). The pore remained closed through the remaining duration of both simulations (e.g., for ∼200 µs in Sim. 2).

To overcome the rapid hydrophobic collapse of the pore and obtain an activated state with a fully open pore, we introduced an activation gate mutation, G178R, which is known to increase the open probability of the homologous KirBac3.1 channel (Bavro et al., 2012). A similar approach has been used by others (Zangerl-Plessl et al., 2020), substituting aspartate rather than arginine for glycine, and their analyses of the resulting G178D mutant, using both x-ray crystallography and MD, arrived at a putative open state of (chicken) Kir2.2. We simulated our G178R pore mutant for a few tens of microseconds (Sim. 3), again in the presence of POPS and with PIP_2_ bound. Concurrent with a slight rotation of the inner TM helix (TM2), both TM helices (TM1 and TM2) splayed, and activation gate residue I177 moved away from the pore axis. Subsequently, proximal residue S174 moved toward the pore axis. The pore of the G178R mutant then settled in into a stable, open conformation within the simulated time (Sim. 3; Fig. 2, A–C; Fig. S1; and Video 1).

**Video 1.** **Intracellular view of pore opening in the G178R activation gate mutant (Sim. 3).** Activation gate residues S174 and I177 are shown as green and cyan spheres, respectively, and PIP_2_ molecules appear as sticks.

During this conformational transition, which we characterize as moving from a pre-opened to a fully open pore conformation, PIP_2_ molecules were bound to the basic residues R78, R80, K183, R186, K188, and K189 of the R/K-rich region near the activation gate. POPS molecules mostly interacted with CTD residues R219 and K220, with another set of R/K residues K62, R65, R78, and R80 of TM1 (a second R/K-rich region), and with the IH, located at the intracellular membrane-water interface where it connects the CTD N-terminus to TM1 (Fig. S2, A–C). Our observations of PIP_2_ binding to two distinct R/K-rich regions are in line with these regions having been previously denoted as primary and secondary lipid-binding sites (Lee et al., 2013). With the negatively charged lipid molecules bound to both of these lipid-binding sites, the CTD moved toward the pore domain by ∼2–3 Å (Fig. S2 D), partly facilitated by the interactions of R219 and K220 with POPS molecules located near the membrane interface (Fig. S2, B and C), and partly by CTD interactions with charged residues of the TM pore domain. The movement of the CTD appears directly coupled to the splaying of helices TM1 and TM2 that resulted in pore opening.

After simulating the opening of the pore, we initiated simulations with a configuration taken from the G178R simulation (Sim. 3) in which the pore was fully open and the G178R mutation was reverted (similar to the approach used by Zangerl-Plessl et al. [2020]). These additional simulations allowed us to study the stability and function of the (R178G-reverted) wild-type (WT) channel by monitoring the cavity hydration of the open pore (Sims. 4–5 and 8), and by recording ion-permeation events from our simulations performed with positive (i.e., depolarizing) transmembrane voltages of 155 ≤ *V* < 375 mV (Sims. 6–12; see Table S1). In Sim. 4, the first 90 μs of simulation revealed a stably open WT pore with no change in pore cavity hydration relative to that observed for G178R (pore closure began to occur after 90 μs and ion permeation completely ceased at 120 μs). Residues S174 and I177 maintained their interchanged positions relative to the pore axis, and the CTD also remained in closer proximity to the pore domain than it was in the pre-opened conformation, ensuring a splay of helices TM1 and TM2 that stabilizes the open pore conformation (Sim. 4; Fig. S2 E).

Simulations of the stably open WT pore under applied voltages of 155 ≤ *V* < 375 mV resulted in steady-state permeation at all voltages (Sims. 8–12; Fig. 2 D and Video 2). The estimated conductance (from Sims. 7–8, performed at 310 mV) was 12.9 ± 2.9 pS. In these two simulations, respectively, 929 and 1,461 potassium ions permeated the channel during 48 µs of simulated time, broadly in line with results from a previous computational study (Zangerl-Plessl et al., 2020), while the experimentally determined conductance ranges from 2–30 pS (Picones et al., 2001). During steady-state permeation, the SF potassium ion occupancy of outwardly permeating ions (i.e., not including ions transiently interacting with the SF from the extracellular side) was 2.87 ± 0.03, with the ions preferentially occupying SF binding sites S1, S2, and S3/S4, as reflected in the ion density profiles across the SF (Fig. 2 E) (We will refer to this measure of occupancy as the “kinetic” ion occupancy to distinguish it from the thermodynamic occupancy observed at equilibrium in, for instance, EM and x-ray structures).

**Video 2.** **Ion conduction across the SF under depolarizing conditions (Sim. 7).** Only SF backbone residues and the cavity controller D173 are shown. Ions and water molecules appear as colored spheres and sticks, respectively.

These simulation results suggest that outward ion permeation (i.e., from the intracellular to the extracellular side of the membrane) in Kir2.2 occurred by a knock-on mechanism in which the following ion configurations were assumed as the ions traversed the SF: S6[S4,S2],S1 → S5[S4,S2],S1 → S5[S3,S2],S0 → S4[S2,S1],S_ext._ (Fig. 2 F). Presence of the charged residue D173 in the pore cavity, along with the positioning of E139 behind the SF, permitted a relatively extensive ion desolvation within the SF during permeation, with a water-to-ion permeation ratio of only 0.22 ± 0.04.

### Long-range electrostatic effect on permeation

**Figure 3.**
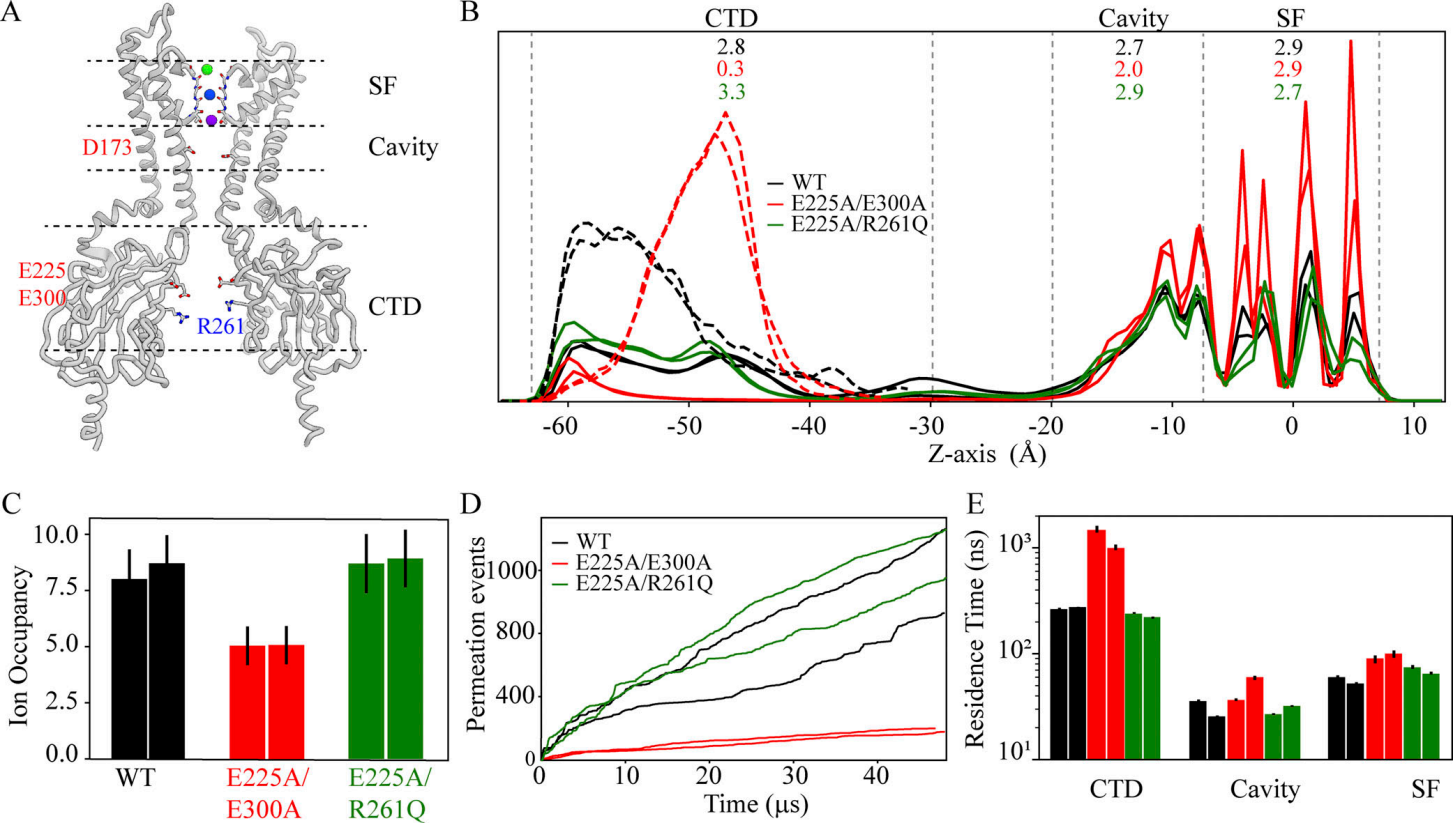
**CTD charge neutralization affects permeation. (A)** Structure of the full-length channel with only two diagonally opposed subunits shown, highlighting various regions within the extended pore. Ions in the SF are individually colored. **(B)** Ion-density profiles (each normalized to 1) with potassium and chloride shown as solid and dashed lines, respectively, across the extended pore in the WT (Sims. 7–8, black) and in the charge density-reduced E225A/E300A and E225A/R261Q double mutants (Sims. 26–27, red; Sims. 28–29, green). The average potassium ion occupancies across the CTD, pore cavity, and the SF are listed for each channel construct. **(C)** Total potassium ion occupancy across the extended pore. **(D)** Ion permeation events as a function of simulation time. **(E)** Ion residence time within the CTD, pore cavity, and SF during permeation in the three channel constructs, with color coding as in B.

During ion permeation, the kinetic potassium ion occupancy across the extended pore was ∼8; ∼2.9 ions occupied the SF, ∼2.7 occupied the pore cavity, and ∼2.8 ions were on average occupying the CTD during permeation (Fig. 3, A–C). Notably, CTD residues E225, R261, and E300 may be important for ion permeation and rectification (Pegan et al., 2005; Fujiwara and Kubo, 2006; Shin et al., 2005; Robertson et al., 2008; Kurata et al., 2007; Chang et al., 2007), as the sidechains of these charged residues line the CTD ion-permeation pathway (Fig. 3 A). We thus decided to investigate the impact of long-range CTD electrostatics on ion permeation and rectification by simulating two CTD mutant pairs: E225A/E300A and E225A/R261Q (Sims. 26–29).

Relative to the simulations of the WT channel, our simulations of the E225A/E300A mutant yielded roughly sixfold lower permeation rates (Fig. 3 D), a reduced potassium ion density across the CTD-pore cavity permeation pathway, and an increased chloride-ion density in the CTD (due to the greater positive-charge density in this mutant resulting from the two charge-neutralized side chains; Fig. 3 B). The permeation mechanism, however, remained unchanged in this mutant (Fig. S3), and the SF steady-state kinetic ion occupancy during permeation was also similar to that of the WT (Fig. 3 B). An order-of-magnitude increase in residence time of the permeating ions within the CTD and a more modest (twofold) increase in the ion residence time in the cavity region (Fig. 3 E), combined with reduced CTD ion occupancy along the permeation pathway, caused the reduced permeation rate for the E225A/E300A mutant at depolarizing potentials. At hyperpolarizing potentials, however, the permeation rates for this mutant were higher than at depolarizing potentials: 0.64 ± 0.04 pA for Sims. 26–27 at 310 mV, compared to −4.44 ± 1.93 pA for Sims. 93–99 at −310 mV (upon SPM unbinding). This effect is known as intrinsic rectification (Fig. 3 D).

Our simulations of the E225A/R261Q mutant—in which a negatively charged residue and a positively charged residue are both charge-neutralized—showed an increased potassium ion density across the CTD and decreased residence time within the pore relative to the E225A/E300A mutant. These two quantities were nearly the same for the E225A/R261Q mutant and the WT, with permeation rates of 4.4 pA for E225A/R261Q and ∼4.0 pA for the WT (Sims. 7–8, 28–29; Fig. 3). Increased potassium ion sequestration by the CTD with elevated bulk potassium concentration, moreover, appeared to mildly influence the permeation rate in the WT (at 310 mV), as the rate doubled with a 2.5-fold increase in bulk potassium concentration: 8.65 ± 1.53 pA at 0.5 M KCl in Sim. 5 vs. ∼4.0 pA at 0.2 M KCl in Sims. 7–8 (see Table S1).

### Role of PIP_2_ in gating

**Figure 4.**
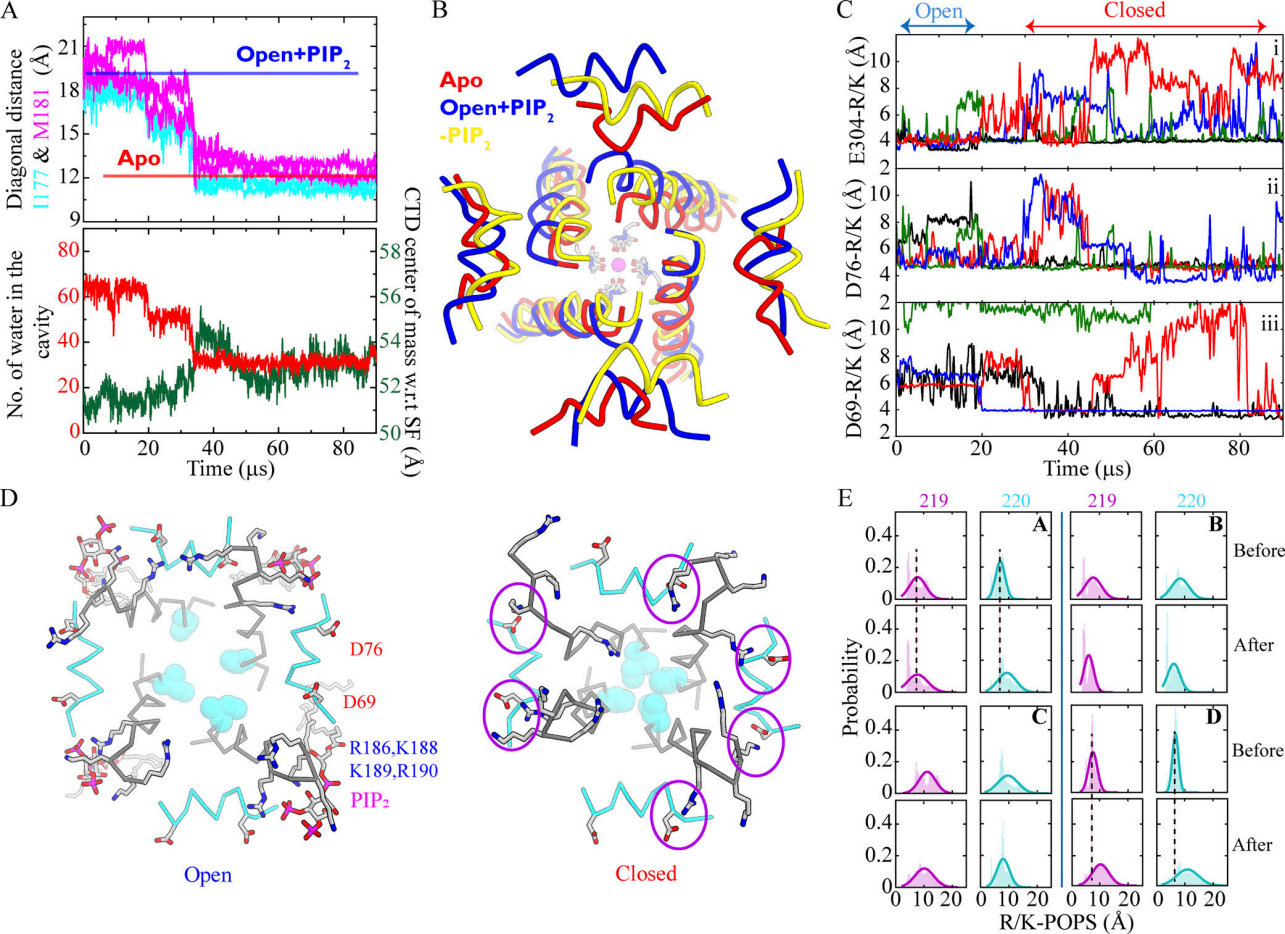
**Channel deactivation in the absence of PIP**_**2**_**. (A)** Channel deactivation and activation gate closure measured by the C_α_–C_α_ diagonal distance at gate residues I177 and M181 (Sim. 13, top), and number of pore cavity water molecules and CTD-SF center of mass separation (Sim. 13, bottom). **(B)** Intracellular view of the inner helix, TM2, and IH in the presence and absence of PIP_2_ (blue, Sim. 4; yellow, Sim. 13), superposed with the PIP_2_-depleted, apo Kir2.2 crystal structure (red, PDB ID 3JYC), with an SF-bound potassium ion in magenta and the C, N, and O atoms of the SF residues shown in gray, blue, and red, respectively. **(C)** Closest atomic distances (per monomer) between any of the TM2 residues of the R/K-rich region (R186:Nζ, K188:Cζ, K189:Cζ, or R190: Nζ) and each of the following residues: (i) E304:Cδ of the G-loop, (ii) D76:Cγ, and (iii) D69:Cγ of the IH. The distance in each monomer is colored individually and the conformation of the channel is indicated by Open and Closed. **(D)** Simulation snapshots revealing proximity between TM2 residues R186, K188, K189, and R190 (gray), and PIP_2_ in the open state (left), and between IH residues D69 and D76 and the R/K-rich region in the absence of PIP_2_ (right). **(E)** Distributions of the CTD R219:Nζ/K220:Cζ–POPS:P distance (per subunit) indicating an altered interaction pattern before and after pore closure and channel deactivation.

Seven simulations (Sims. 13–19) of the WT channel without PIP_2_ bound, initiated from a channel conformation in which the pore was fully open (Sim. 3; see Table S1), were carried out to investigate the effect of PIP_2_ depletion on the stability of the open-pore conformation. Of these seven simulations, five (Sims. 13–16 and 18) resulted in faster pore closure compared to simulations with PIP_2_ (Sims. 4–6 and 11), as well as a decrease in the diagonal inter-subunit distance at the activation gate and a decrease in pore-cavity hydration. These five simulations each arrived at a pore conformation resembling that of the apo-like, PIP_2_-depleted crystal structure, with a C_α_ RMSD of 2.0 ± 0.16 Å (PDB ID 3JYC; Tao et al., 2009; Fig. 4, A and B; Sims. 13–19; Video 3). The median pore-closure time in these PIP_2_-depleted simulations was 58 µs, compared to >120 µs with PIP_2_ bound (Fig. S6; Sims. 4–6, 11, and 13–19), consistent with the agonizing role of PIP_2_ in Kir2.2 channel function.

**Video 3.** **Pore closure in the absence of PIP**_**2**_ **(Sim. 13).** Intracellular view of the pore domain with activation gate residues S174 and I177 shown as green and cyan spheres, respectively.

As the pore underwent closure, the CTD dislodged from the TM domain (Fig. 4 A), while the collapse of the TM helices at the activation gate permitted the IH to move towards the pore axis (Fig. 4 B and Video 4). In the absence of PIP_2_, the TM1/TM2-located R/K-rich region instead interacted with D69 and D76 on the IH, and the distance distribution of this set of salt bridges indicates that these distances all shortened upon pore closure (Fig. 4, C and D). In these PIP_2_-depleted simulations, we also observed that salt bridges formed between E304 on the G-loop—a known cytoplasmic gating region (Pegan et al., 2005)—and residues of the R/K region, while interactions of CTD residues R219/K220 with the negatively charged POPS lipids were lost as the CTD separated from the TM domain (Fig. 4, C and E).

**Video 4.** **Conformational changes of the IH (blue) and TM2 (gray) during pore closure in the absence of PIP**_**2**_ **(Sim. 13).**

Having observed that IH residues D69 and D76 appeared to be involved in Kir deactivation upon PIP_2_ removal—presumably by stabilizing a deactivated state of the channel—we performed additional simulations with a D69A/D76A double mutant (Sims. 13–25). These simulations resulted in a stable, open, and conducting channel, even in the absence of PIP_2_ (Sims. 20–25), and the median pore closure time of the double mutant was comparable to that of PIP_2_-bound WT (110 and 120 µs, respectively; Fig. S6).

### SPM binding and inward rectification

**Figure 5.**
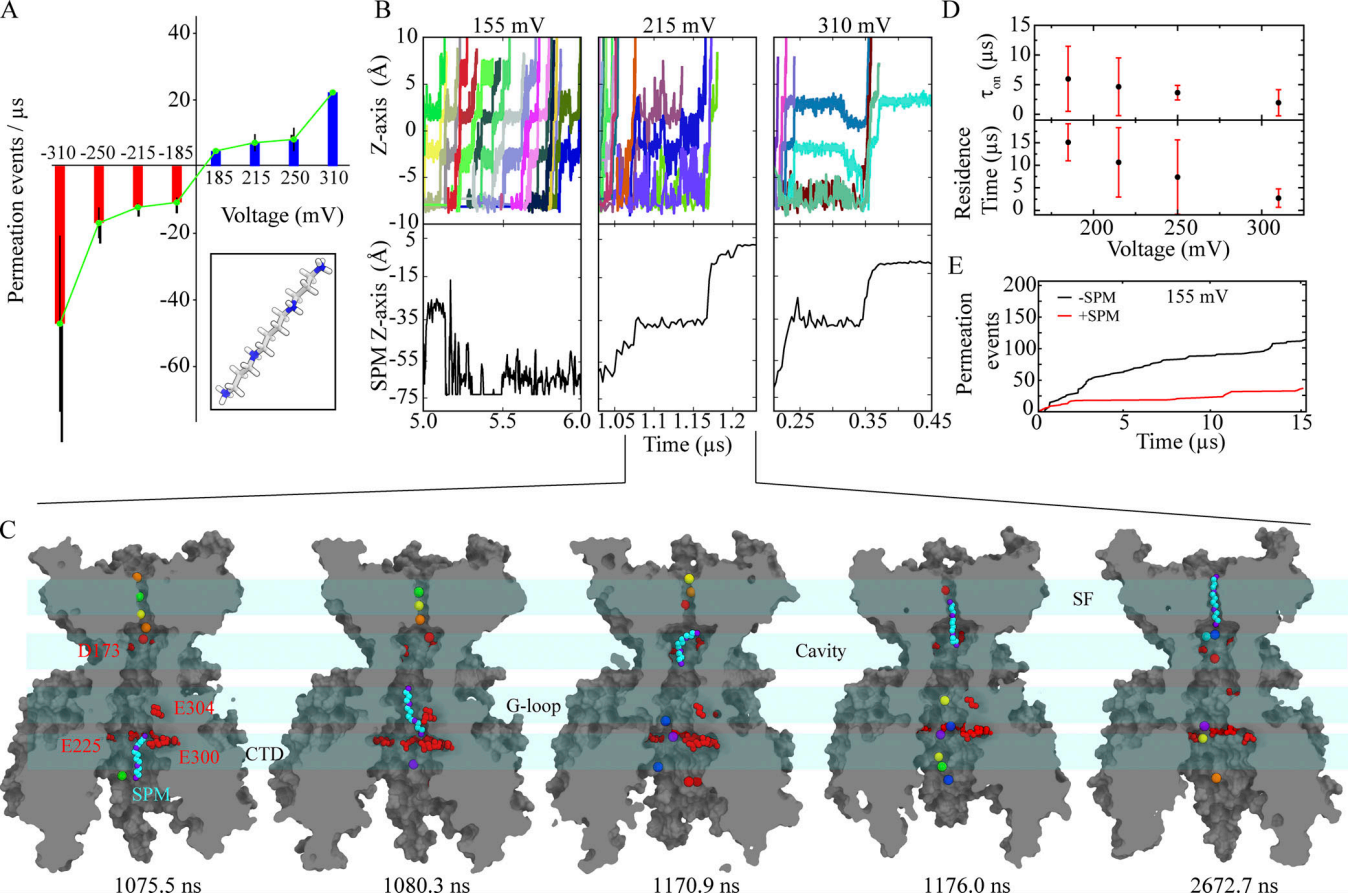
**SPM binding and pore block. (A)** Ion permeation events per unit time under depolarizing (Sims. 31–46, blue) and hyperpolarizing (Sims. 51–58, red) conditions, and in the presence of SPM. Inset: Molecular structure of SPM. **(B)** Ion transitions across the SF (top) and SPM position relative to the D173 center of mass (C_α_ atoms; bottom) at three different depolarizing voltages (Sims. 48, 39, and 32). **(C)** Simulation snapshots (Sim. 39) revealing sequential SPM (in cyan) binding resulting in pore block, with potassium ions individually colored. **(D)** Voltage-dependent SPM binding (Fig. S4 A) and SPM residence times at the pore cavity-SF region (bottom; Sims. 31–46). **(E)** Ion permeation events as a function of simulation time in the presence (red, Sim. 47) and absence (black, Sim. 12) of SPM at low voltage (155 mV), where SPM is only weakly bound, and mostly to the CTD.

We simulated SPM binding using the fully activated Kir2.2 conformation and observed SPM binding to the open pore, and subsequent pore block, at depolarizing potentials (155 ≤ *V* < 310 mV; Sims. 30–50), but not at hyperpolarizing potentials (−310 ≤ *V* < −155 mV; Sims. 51–59). This observation is in line with the inward rectification known to occur physiologically with endogenous polyamines (or magnesium ions) blocking the channel. The SPM-mediated pore block manifested in a decrease in ion permeation events at depolarizing voltages (Sims. 30–50; Fig. 5 A and Fig. S4 D). Depending on the magnitude of depolarizing voltages, we observed three distinct SPM-binding scenarios: (1) at low applied voltage (*V* < 185 mV), SPM binding was predominantly restricted to CTD; (2) at intermediate voltages (185 ≤ *V* < 215 mV), SPM binding to the pore cavity and the lower SF region and persistent pore block occurred; (3) at high voltages (*V* ≥ 250 mV), SPM resided only transiently at the pore cavity and lower SF region before translocating across the SF to exit on the extracellular side, transiently triggering ion permeation (Fig. 5, A and B; and Fig. S4 A). At all depolarizing potentials of 185 mV or greater, SPM binding resulted in displacement of 5–6 ions located within the TM pore, including those bound to the SF (Fig. 5, B and C).

From these SPM-binding simulations, we identified five distinct channel regions that preferentially accommodated SPM: (1) a region of the CTD near E225 and E300 rich in negative charge; (2) the G-loop near E304; (3) the pore cavity; (4) a region above the cavity-located D173; and (5) the SF (Fig. 5 C, Fig. S4 C, and Video 5). From these simulations, we estimated a voltage-dependent SPM-binding time constant (τon). At high depolarizing voltages (*V* ≥ 250 mV), SPM binding was faster (τon values were in the range 1.9–3.5 µs) than at intermediate voltages (185 ≤ *V* < 215 mV), where τon values were in the range of 4.6–6.0 µs (Fig. 5 D and Fig. S4 A). These on-rates are two to three orders of magnitude faster than what has been observed in experiments at physiological voltages of 60 ≤ *V* ≤ 100 mV (Kurata et al., 2007), likely due to the higher, nonphysiological voltages applied in our simulations. The SPM residence time within the pore was also voltage-dependent, with the longest residence at intermediate voltages and shortest residence at high voltages due to fast translocation across the entire pore, including the SF (Fig. 5 D and Fig. S4 A).

**Video 5.** SPM binding and pore block under depolarizing conditions (Sim. 39).

In the SPM-binding simulations, a ring of negatively charged CTD residues composed of the four E225 and four E300 residues in the tetramer was observed to act as an electrostatic sink that sequestered the positively charged SPM to the CTD, and a high SPM density around this ring center was observed in all our SPM-binding simulations (Fig. S4 C). The CTD region appeared, however, to harbor only a shallow SPM-binding site, with several SPM binding and unbinding events occurring in this region (Fig. S4 B). During simulations in which the CTD ring was charge-neutralized by mutating the E225 and E300 residues to alanine, no SPM binding or pore block occurred (Sims. 91 and 92); rather, SPM transiently bound to a peripheral CTD region (Fig. S4, B and C). These observations are in accordance with multiple experimental findings suggesting these charged CTD residues influence the kinetics of SPM binding and unbinding (Fujiwara and Kubo, 2006; Kurata et al., 2007; Kubo and Murata, 2001).

**Figure 6.**
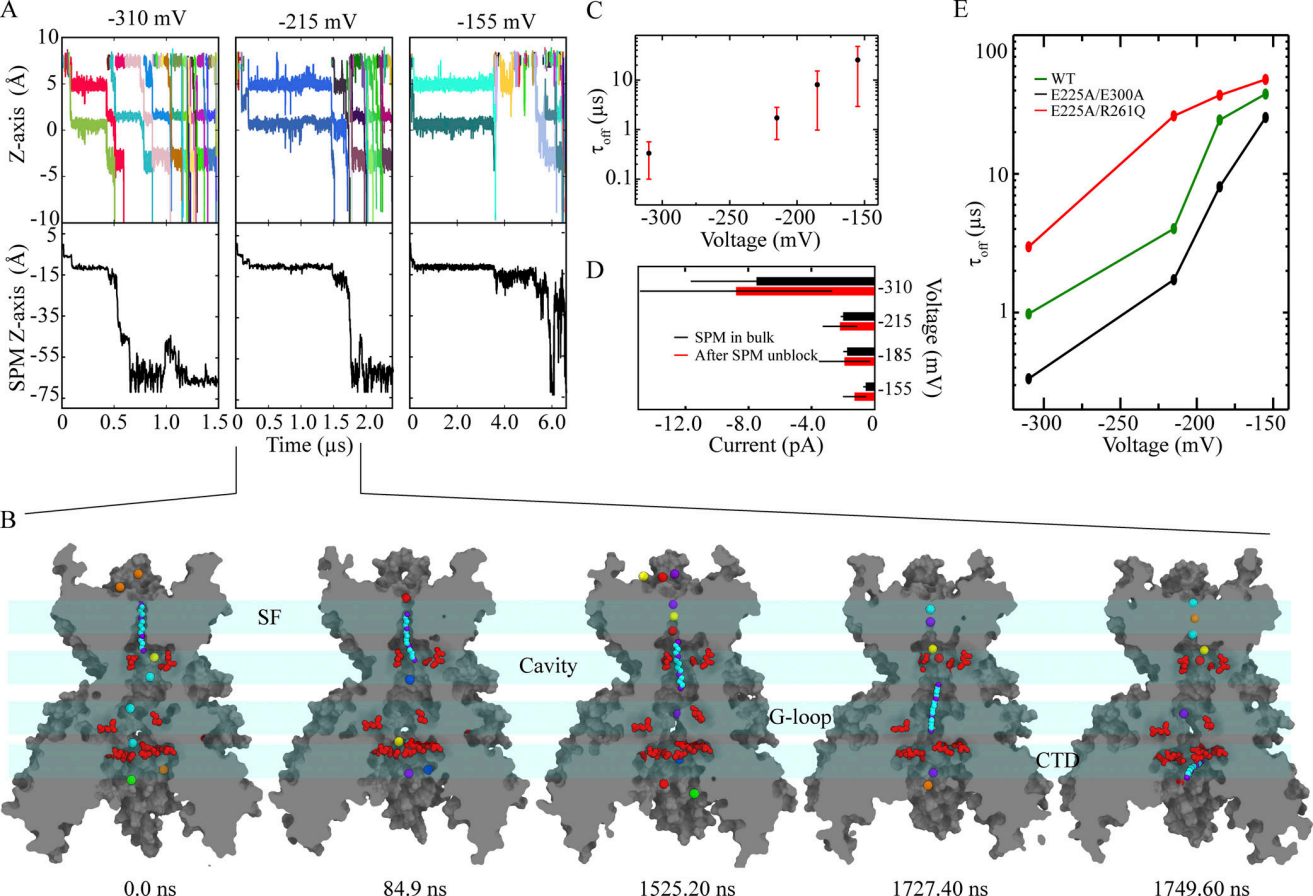
**SPM unbinding under hyperpolarizing conditions. (A)** Ion transitions across SF (top) and SPM position relative to the D173 center of mass (C_α_ atoms; bottom) at three different hyperpolarizing voltages (Sims. 63, 69, and 90). **(B)** Simulation snapshots (Sim. 69) revealing sequential SPM (cyan) unbinding, with the individual residues represented as in Fig. 5 C. **(C)** Voltage-dependent SPM unbinding times (Sims. 61–90), with unbinding identified when the distance between SPM and the D173 center of mass (C_α_ atoms) exceeded 20 Å (30-ns running medians). **(D)** Current vs. hyperpolarizing voltage with SPM present in bulk solution (Sims. 51, 52, 55–59; black), and after SPM unbinding from the pore cavity-SF region (Sims. 61–81, 83–90; red). **(E)** SPM-unbinding times in WT (Sims. 61–81, 83–90; black) and in the negative charge density-reduced double mutant E225A/E300A (Sims. 93–120, red) and in E225A/R261Q (Sims. 121–148, green).

We simulated time- and voltage-dependent SPM unbinding starting from a SPM-bound, pore-blocked state (Sim. 30; see Table S1) under hyperpolarizing conditions (−155 ≤ *V* < −310 mV; Sims. 60–90), and observed voltage-dependent SPM unbinding from its main binding site, which extends from D173 in the pore cavity to the lower SF region (Fig. 6, A and B; and Video 6). Inward potassium permeation occurred upon relief from the SPM block (Fig. 6 A), and the SPM-unbinding times were shorter at stronger hyperpolarizing voltages: 0.33 ± 0.23 µs (at −310 mV) vs. 8.1 ± 7.1 µs (at −185 mV; Fig. 6 C). The post-SPM-unbinding ion permeation rates were comparable to the rates we observed in our simulations at hyperpolarizing voltages and to the rates observed in simulations performed in the presence of non-binding, bulk-located SPM (Sims. 51–59; Fig. 6 D).

**Video 6.** SPM unbinding and relief from pore block under hyperpolarizing conditions (Sim. 69).

Additional SPM-unbinding simulations were conducted using the E225A/E300A double mutant (at −310 ≤ *V* < −155 mV; Sims. 93–120) and the E225A/R260Q double mutant (at −310 ≤ *V* < −155 mV; Sims. 121–148) to establish a correlation between SPM-unbinding times and CTD charge density. In the simulations of the E225A/E300A double mutant with a charge-neutralized CTD ring (Sims. 93–120), the SPM-unbinding times were longer, suggesting that E225 and E300, important for recruiting SPM, also play a key role in ensuring rapid SPM unbinding and relief from pore block (Fig. 5 E and Fig. S5). A role in unbinding is further supported by results from simulations of an E225A/R261Q double mutant (Sims. 121–148), constructed to compensate for the loss of CTD negative-charge density (i.e., due to the E225A mutation). The E225A/R261Q double mutant exhibited, at −310 ≤ *V* < −155 mV, SPM-unbinding times and ion-permeation rates comparable to those of the WT (4.4 vs. 4.0 pA in WT at −310 mV; Sims. 28 and 29; Fig. 3 B, Fig 5 E, and Fig. S5).

We next conducted simulations (Sims. 149–152) designed to study the effects of short-range electrostatics on SPM block. Specifically, we simulated the influence of the protonation state (i.e., charge) of cavity-located residue D173 by deprotonating all four D173 residues (rather than the two deprotonated residues in previous simulations). The ion occupancy of the pore cavity increased in Sim. 149 from ∼2 with two D173 residues deprotonated to ∼4 with all four D173 residues deprotonated; the higher ion occupancy hampered SPM entry into the pore cavity and subsequent translocation toward the SF (in Sims. 150–152). The voltage dependence of SPM binding shifted accordingly toward more positive voltages; only at voltages above 250 mV was SPM entry into the pore cavity and translocation toward the SF observed (Sims. 150–152). The SPM residence time in the pore cavity and SF region was also slightly longer with four D173 residues deprotonated compared to two (14.8 vs. 7.4 µs, respectively, at 250 mV), which is unsurprising given the higher negative charge density in the fully deprotonated configuration (Fig. S4 E).

Our SPM binding and unbinding simulation results suggest that voltage-dependent SPM unbinding is indeed responsible for Kir2.2 inward rectification, and that the rectification controller, D173, and its protonation state controls SPM entry into the pore cavity and SPM binding to the pore cavity and lower SF region.

## Discussion

### Lipid-dependent gating

In an attempt to obtain a fully activated Kir2.2 conformation with a stably open and conducting pore, we initiated simulations from the crystal structure of cKir2.2 bound by PIP_2_ (Hansen et al., 2011) in the presence of POPS lipid molecules (the latter lipid is known to increase the Kir2.2 open probability and single-channel conductance; Lee et al., 2013; Choe et al., 2001; Cheng et al., 2011; Lee et al., 2016). Although these two lipid molecules bound the primary and secondary lipid-binding sites, respectively, the pore underwent closure (Sims. 1 and 2; Fig. 2 A).

The ability of the channel to undergo closure despite being agonized by lipid molecules appears to be in line with data from single-channel electrophysiological measurements that suggest strong inward rectifier channels (e.g., hKir2.1) can assume a closed, deactivated state, with the open probability increasing in the presence of PIP_2_ (Panama et al., 2010). These experimental results appear broadly consistent with our simulations, in which we observed fast pore closure of what likely represents a preactivated state of cKir2.2 (Sim. 2; Fig. 2 A; and Fig. S1, A and B). Unfortunately, our simulations failed to produce pore-opening of the WT channel in the presence of PIP_2_ (regardless of the additional presence of POPS) on the simulated timescales, which might have been too short compared to the experimental timescale (rate) of pore opening. Our inability to observe pore opening of the WT channel in simulations might also have been due to shortcomings of the force field used.

To simulate channel activation, we took advantage of the experimental finding that in the bacterial KirBac3.1 channel, a homolog of Kir2.2, mutation of a serine residue to arginine at the main activation gate (S129R) was shown to cause gain of function (Paynter et al., 2010), and a crystal structure of this mutant revealed a C_α_–C_α_ distance at the activation gate region (measured at Y132) of 17 Å (PDB ID 3ZRS; Bavro et al., 2012). More recently, a crystal structure of cKir2.2 with the S129R-corresponding mutation (G178D) was reported (PDB ID 6M84; Zangerl-Plessl et al., 2020), and the introduction of G178D similarly resulted in an opened channel, featuring a substantially widened pore; the C_α_–C_α_ distance at the activation gate (measured at I177) is 12.9 Å, whereas the corresponding distance in the WT PIP2-bound cKir2.2 crystal structure is only ∼10 Å. We thus introduced G178R in our simulations of cKir2.2, which resulted in an open-pore conformation with an I177 C_α_–C_α_ distance of 16.5 ± 0.8 Å (Sim. 3; Fig. 2 A). During the more extensive pore opening caused by the G178R mutation, S174 and activation gate residue I177 interchanged positions—I177 moved away from the pore axis, while S174 simultaneously moved towards it—as a result of substantial movement (i.e., rotation) of the inner (TM2) helix at the activation gate region (Fig. 2, A–C).

The presence of PIP_2_ at the primary lipid-binding site near helices TM1 and TM2, and of negatively charged POPS lipid molecules at the secondary binding site near the IH, were both observed to be important for bringing the CTD nearer to the TM region, thereby increasing the stability of an activated conformation (Fig. S2). This is consistent with previous suggestions that negatively charged PIP_2_ and POPS lipid molecules appear to act as positive allosteric modulators (Lee et al., 2013; Choe et al., 2001; Cheng et al., 2011; Lee et al., 2016).

Having simulated the WT channel with its pore in the fully open conformation (Sims. 4–12), we decided to study potential conformational changes that would occur upon removal of PIP_2_ from the simulated membrane. Within a few tens of microseconds of simulated time (Sims. 13–19), we observed complete closure of the pore (Fig. 4, A, B, and D). The resulting conformation of the channel with the closed pore indeed resembled that of the apo-like (i.e., PIP_2_-depleted) crystal structure, with a TM2 C_α_ RMSD of 2.0 ± 0.16 Å (Fig. 6 B; PDB ID 3JYC; Tao et al., 2009). This suggests that our simulations, started from a fully activated state (obtained with the G178R mutant), were able to produce a closed state similar to what has been observed experimentally (Lee et al., 2016) when PIP_2_ is no longer agonizing the channel.

Upon pore closure, the CTD dislodged from the TM domain, as interactions between the positively charged CTD residues and the negatively charged lipids—interactions essential for this tethering—were lost. At the same time, alternate interactions between the R/K-rich region on the TM domain and residues D69 and D76 on the IH were formed instead (Fig. 4, A, C, and E). Notably, by comparing our closed state Kir2.2 conformation with the apo, PIP_2_-depleted Kir2.2 closed-state crystal structure, we also found residue D76 to be positioned in close proximity to the TM R/K-rich region (D69 was unresolved in the apo crystal structure), suggesting that their mutual interactions are important for gating and stabilization of the closed state. In the closely related Kir1.1 channel, a hydrogen bond between the ɛ-nitrogen of K80 (located on TM1 near the IH) and the backbone carbonyl oxygen of TM2 residue A177 appears to stabilize the closed state (Rapedius et al., 2007). This hydrogen bond may thus play a role in gating of Kir1.1 that is related to the role of the salt-bridge interactions between Kir-conserved IH residues D69 and D76 and the TM R/K residues in the deactivated, closed-pore Kir2.2 conformation observed in our simulations.

The conformational changes observed upon channel deactivation suggest a potential Kir activation mechanism: during activation, the TM R/K-rich region at the activation gate (i.e., the primary lipid-binding site) latches onto PIP_2_, thereby displacing Kir-conserved IH residues D69 and D76, residues that form salt bridges with the same R/K region in the deactivated conformation. This exchange of interactions enables the IH to slide away from the pore axis and CTD residues R219 and K220, thus forming a secondary lipid-binding site that can interact with negatively charged POPS lipid molecules, as observed in our pore-opened, activated Kir2.2 conformation (Fig. S2 C). This enables the CTD to move further toward the pore domain—a movement facilitated by the TM helices becoming more flexible—and the IH is displaced (concertedly or sequentially) from the pore axis upon opening of the activation gate. Recent structural work has supported the functional importance of the IH in gating of hKir6.2 (Rapedius et al., 2007), revealing a displacement of IH during gating that is similar to the displacement we observed for Kir2.2.

**Figure 7.**
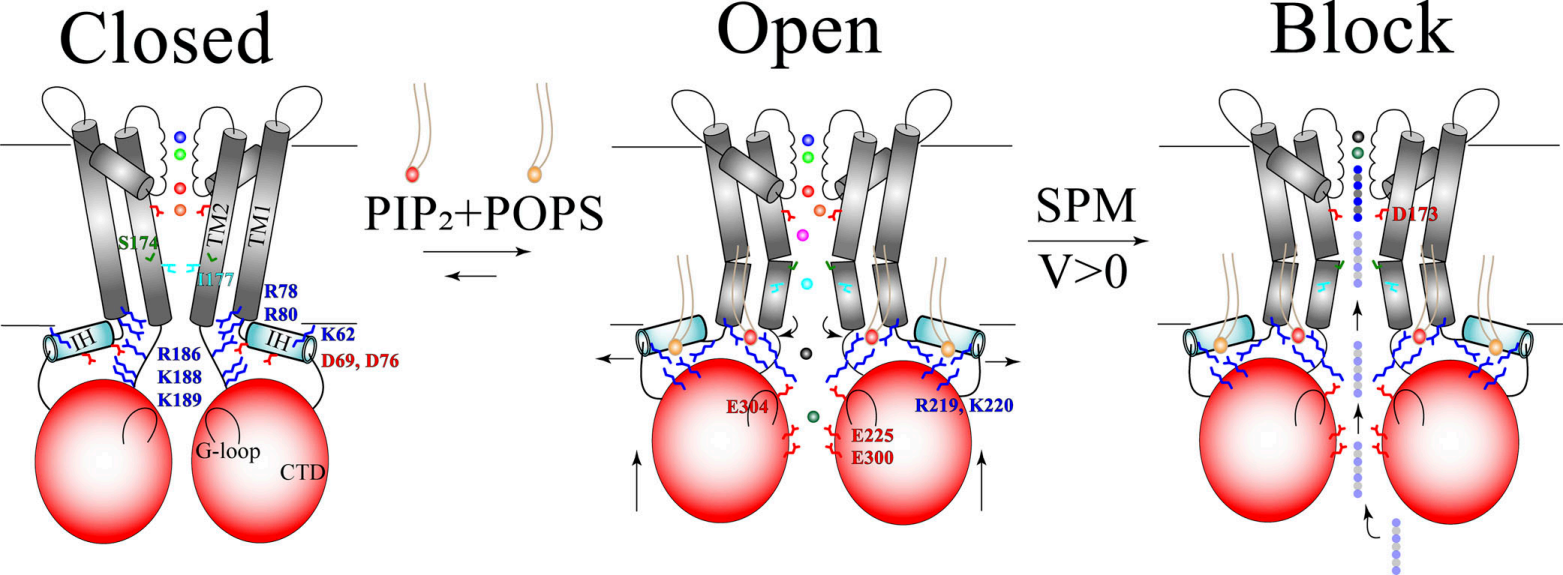
**Model of lipid-dependent activation mechanism and SPM pore block.** Closed conformation: In the absence of negatively charged lipids, positively charged R/K residues near the activation gate engage in salt bridge interactions with negatively charged IH residues and TM1 backbone residues, thereby stabilizing the closed, deactivated channel conformation. Open conformation: In the presence of negatively charged PIP_2_ and POPS lipid molecules in the membrane, three key sequential structural changes lead to pore opening and channel activation: (1) the R/K-rich region loses interaction with negatively charged IH residues in exchange for interactions with PIP_2_ molecules at the primary lipid-binding site, which leads to disengagement of the IH from the pore domain, allowing the IH to move radially away from the pore axis; (2) the CTD then moves towards the pore domain and CTD residues R219 and K220 of the secondary lipid-binding site interact with POPS lipid molecules, permitting G-loop residue E304 to interact with the R/K-rich region; and (3) slight rotation and splaying of inner TM helices occurs, leading to pore opening. Blocked conformation: Under depolarizing conditions (*V* > 0), SPM (grey/blue) is recruited by the CTD and sequentially translocates toward the pore cavity-SF region, where it displaces approximately five cavity/SF-bound potassium ions as it binds above the D173 rectification controller, blocking the ion permeation pathway.

This conformational rearrangement enables TM2 to move away from the pore axis, resulting in widening of the main activation gate and pore opening (Fig. 7). As a consequence of the additional CTD displacement, a secondary cytoplasmic gate constituted by the G-loop also opens, as G-loop residue E304 can interact with the TM R/K-rich region in this conformation (Fig. 4 C). This interaction pushes the G-loop away from the pore axis, reflected in a change of the C_α_–C_α_ distance from 13.9 Å at the narrowest region in the closed-state Kir2.2 apo crystal structure (PDB ID 3JYC) to 16.4 Å in the fully activated state observed in our simulations, suggesting a critical role for E304 in Kir2.2 gating (as previously proposed by Pegan et al. [2005]).

Simulations performed with Kir-conserved IH residues D69 and D76 both mutated to alanine resulted in increased stability of the open channel, even in the absence of PIP_2_ (Sims. 20–25); the median pore closure time found in these simulations was comparable to that observed in our simulations of the PIP_2_-bound WT structure (Fig. S6). Functional analysis of the corresponding IH residues in the Kir6.2 channel has highlighted the importance of these aspartate residues, which tether the TM and CTD domains (Li et al., 2013) as gating modulators. Despite the observation that the D69A/D76A mutant showed a higher propensity to remain in an open, conducting conformation than the WT channel (presumably due to the reduced negative-charge density in the double mutant), lower currents were observed for the double mutant than for the WT (Table S1; 1.67 ± 0.62 pA for D69A/D76A [Sims. 20−25] versus an average of 4.1 ± 0.87 pA for the seven simulations of the WT [Sims. 13–19]). Reduced negative-charge density might be one plausible reason for the loss of function observed in experiments with D58A or D65A charge-neutralizing mutations at the corresponding positions in hKir6.2 (Li et al., 2013).

The importance of the IH domain in channel function is reinforced by the fact that several IH residues are also involved in channelopathies (Hibino et al., 2010). By way of example, hKir2.1 missense mutations D71V and D78Y—at residues that correspond to D69 and D76 in cKir2.2—cause loss of function and a clinical condition known as Andersen’s syndrome (Plaster et al., 2001; Decher et al., 2007). This is likely because interactions between these bulky, neutral mutant residues and TM2 decrease the current by stabilizing the closed state. It is also possible that these bulky and neutral sidechains destabilize the secondary structure of the amphipathic IH helix, which thereby loses its ability to function as a modulatory gating element.

### Permeation

Having successfully obtained an activated Kir2.2 conformation with a fully open and ion-conducting pore, we were able to simulate and observe steady-state ion permeation (Sims. 7–12). As previously observed in MD simulations of related potassium-selective ion channels (Jensen et al., 2013), however, the simulated permeation rates are severely underestimated at experimentally accessible and physiologically relevant voltages due to various shortcomings of the force field. This generally necessitates the use of artificially high voltages to achieve the number of permeation events required to draw conclusions from the simulations (e.g., to deduce a potential permeation mechanism that may still apply in the absence of this voltage shift; Fig. 2 D; Jensen et al., 2013).

On aggregate, we observed a large number of permeation events at different transmembrane potential differences (Table S1), allowing us to determine a kinetic SF ion occupancy of 2.87 ± 0.03, which is marginally higher than the occupancy of 2.6 ± 0.2 observed in our related simulation study of the voltage-gated Kv1.2/2.1 potassium channel (Jensen et al., 2013). A relatively low water co-occupancy in the SF of 0.22 ± 0.04 is, by way of contrast, lower than what we observed in our Kv1.2/2.1 simulations (0.5 ± 0.2). This difference can be attributed to the relatively higher SF ion occupancy during permeation in Kir2.2.

The Kir2.2 ion-permeation mechanism appears to be a knock-on type mechanism, similar to that proposed to operate in voltage-gated potassium channels such as Kv1.2/2.1 (Jensen et al., 2013); in Kir2.2, however, one water molecule co-permeates with about four permeating potassium ions (Fig. 2 E; Jensen et al., 2013; Zangerl-Plessl et al., 2020; Bernsteiner et al., 2019). We believe that near-identical knock-on permeation mechanisms exist in most potassium-selective channels, and that these mechanisms mainly differ in the amount of co-permeating water, all depending on the local electrostatic potential within and near the SF, which in Kir2.2 appears to allow for relatively extensive ion dehydration (as has been observed previously; Zangerl-Plessl et al., 2020). A mechanism with a substantial amount of co-permeating water and less pronounced ion desolvation may predominate in some potassium channels (e.g., as we found for the Kv1.2/2.1 channel using a similar setup; Jensen et al., 2013), whereas a mechanism with more extensive ion desolvation may predominate in channels where the water-to-ion permeation ratio seems to be lower (e.g., in the KcsA channel; Jensen et al., 2013; Furini and Domene, 2009; Köpfer et al., 2014).

A greater amount of co-permeating water was observed in our Kir2.2 simulations than in shorter simulations of the same protein that were reported previously (Zangerl-Plessl et al., 2020); aside from the differing simulation timescales, these contrasting results may have also resulted from differences in the simulation setups used in these studies, including the selection of force fields. The preferred location of water during permeation may also differ between different potassium channels. In Kv1.2/2.1, for example, water was found interspersed between partially hydrated ions (Jensen et al., 2013), whereas in our simulations of Kir2.2, though the ions in binding sites S4 and S3 (closest to the pore-cavity) were partially hydrated, the ions in the other binding sites were essentially dehydrated (Fig. 2 E), as has been observed previously (Zangerl-Plessl et al., 2020; Furini and Domene, 2009; Köpfer et al., 2014).

A more extensive ion desolvation is generally observed in potassium channels that possess greater negative-charge density near the SF. In Kir2.2, for example, this is due to the presence of E139 (interacting with R149 behind the SF), and D173 in the TM pore cavity (Tao et al., 2009; Hansen et al., 2011); in KcsA, this is due to E71 and D80 (both located behind the SF; Zhou et al., 2001). Differences in the ion configuration that awaits the rate-determining knock-on from an incoming ion can likely be attributed to the presence of these negatively charged residues.

### CTD impact on ion permeation

The potassium ion occupancy of the CTD seems to influence the kinetics of ion translocation across the cavity and SF. It is conceivable that the CTD acts like an electrostatic sink, recruiting permeating potassium ions and positively charged pore-blockers such as magnesium ions and SPM. In our WT Kir2.2 simulations, the potassium ion occupancy of the CTD was almost the same as for the pore cavity (Fig. 3 B), due to the substantial and comparable negative charge density in both regions. We then conducted simulations of double mutants in which key negatively charged CTD residues—E225, R261, and E300, all known to influence ion permeation and channel block (Fujiwara and Kubo, 2006; Robertson et al., 2008; Yang et al., 1995)—were charge-neutralized. In the case of E225A/E300A (Sims. 26 and 27), for example, we observed an approximately sixfold decrease in permeation rate compared to that of the WT, but with the same permeation mechanism as for the WT (Fig. 3 D and Fig. S3).

The sixfold reduced permeation rate in E225A/E300A resulted from rarer (i.e., rate-limiting) formation of the knock-on intermediate, which can be attributed to a relative (to the WT) decrease in ion occupancy within the CTD leading to longer residence time of the translocating ions in the pore cavity (Fig. 3). This finding indicates that the rate of ion permeation is highly influenced by the CTD, an idea that is consistent with experimental observations that neutralizing these negatively charged CTD residues decreases outward ion conductance (Fujiwara and Kubo, 2006; Yang et al., 1995; Zhang et al., 2004). Negatively charged CTD residues thus appear responsible for sequestering potassium ions in Kir2.2, and for recruiting potassium ions to the protein–membrane interface region to destabilize the potassium ions residing in the pore cavity. This recruitment leads to more frequent knock-on in the SF by an incoming ion and, ultimately, to a higher ion permeation rate.

It should be noted that semi-quantitative mechanistic assertions based on MD simulations—including our proposed gating mechanism, and more generally, our conclusions about lipid-dependent activation of Kir2.2—remain associated with unavoidable uncertainties, as simulation results can depend strongly on the details of the force fields used (Jensen et al., 2012; Furini and Domene, 2009; Köpfer et al., 2014; Bernèche and Roux, 2001). Additionally, more quantitative measures often have systematic errors in MD simulations; permeation rates, for example, are generally underestimated (Jensen et al., 2013). Over time, however, it is possible that polarizable force fields and more accurate membrane models will improve the quantitative accuracy of MD simulations of ion channel function.

### CTD impact on intrinsic rectification

In our simulations of the E225A/E300A double mutant, an increased chloride density along the CTD pore was observed at both depolarizing and hyperpolarizing potentials. The increased density led to a decreased outward permeation rate at depolarizing potentials (Fig. 3 B), but did not impact the inward permeation rate under hyperpolarizing conditions (Table S1; Sims. 26–27, 93–99). This indicates that at depolarizing potentials, the increased chloride density in the CTD constitutes a rate-limiting barrier for outward permeation, making the E225A/E300A mutant inwardly rectifying. These observations appear to be in line with the experimentally observed intrinsic inward rectification in E225A, which can be mitigated by introducing a compensatory CTD mutation, R261Q. This effect of E225A might be due to the larger positive-charge density within the CTD (compared to WT) leading to accumulation of chloride ions along the ion-permeating CTD pore, thereby resulting in an increased barrier for outward potassium ion permeation (Fujiwara and Kubo, 2006). Similarly, when a positively charged residue is introduced in ROMK at the position of the rectification controller (N171R), intrinsic inward rectification has been observed due to increased positive-charge density within the pore cavity region hampering outward ion permeation (Lu and MacKinnon, 1994).

Consistent with the mitigating effect we observed on the permeation rate in the E225A/R261Q mutant, further simulations of this mutant showed an increase in potassium ion occupancy along the CTD pore and a decrease in ion residence times across the pore overall, resulting in ion permeation rates comparable to those of the WT (Fig. 3 and Table S1; Sims. 28–29). These observations together suggest that the combination of charged residues in the CTD is fine-tuned to ensure a steady-state CTD potassium ion occupancy that maximizes the ion permeation rate of Kir2.2.

### Rectification due to SPM

In our simulations of SPM binding to the activated Kir2.2 conformation (Sims. 30–50), we observed SPM translocate (under depolarizing conditions) along the extended pore from the CTD—initially recruited by residues E225 and E300 (Fig. S4)—to the pore cavity and SF region, where SPM ultimately bound between D173 and the SF. This binding resulted in occlusion of the SF and blockage of the pore at voltages of ≥185 mV (Figs. 5 and 7). During SPM binding, five to six cavity- and SF-located potassium ions were displaced (Fig. 5, B and C), consistent with the displacement of four to six elementary charges observed experimentally (Guo and Lu, 2003; Pearson and Nichols, 1998). These observations imply that the charge movement associated with the SPM block is predominantly due to the displaced potassium ions. They are also consistent with the previous observation that increased external potassium ion concentration shifts voltage-dependent SPM binding toward more positive potentials as it becomes increasingly difficult for SPM to outwardly displace SF-bound potassium ions (Lopatin and Nichols, 1996). The cavity and SF ion occupancies of 4.2 and 2.9, respectively, observed in our simulation with all four D173 residues deprotonated (Sim. 149), are too high to be compatible with the displacement of four to six elementary charges observed in experimental studies of voltage-dependent SPM binding (Guo and Lu, 2003; Pearson and Nichols, 1998), as SPM would be expected to displace all ions in the cavity and the SF. This suggests that only two D173 residues, on average, are deprotonated under physiological conditions.

At lower voltages of <185 mV, no persistent SPM block was observed in our simulations, yet the permeation rates did decrease (Fig. 5 E and Fig. S4 B). This result is consistent with single-channel experiments at low experimental voltages that showed decreased current amplitudes without any change in the open probability, suggesting that only the ion permeation rate was influenced by weakly bound SPM. At stronger depolarizing conditions (*V* ≥ 185 mV), the experimentally observed reduction in single-channel current amplitudes was, by contrast, attributed to rapid block of the open pore caused by SPM entering deeply into the TM pore domain (Xie et al., 2002).

In our simulations of the E225A/E300A double mutant (Sims. 91 and 92), reduction of the CTD negative-charge density resulted in a channel that was unable to recruit SPM into the extended pore, where initial recruitment of SPM would typically occur; only transient binding to the CTD periphery was observed, even at the strongest depolarizing conditions tested (*V* ≥ 310 mV; Fig. S4 B). SPM binding to the CTD appears to be the first of the sequential SPM-binding steps that ultimately leads to pore block, as SPM traverses deep into the TM pore and its cavity, and eventually binds between the cavity-located rectification controller D173 and the SF. This model is in line with experimental observations of reduced SPM binding to an E225A/E300A-homologous mutant, E224G/E299S Kir2.1 (which is also partly deficient in CTD negative-charge density; Guo and Lu, 2003; Robertson et al., 2008; Yang et al., 1995; Xie et al., 2002).

Our simulation results suggest that D173 is essential for strong SPM binding (Fig. 5), which is consistent with a previous report that a D173-neutralizing mutation, D173N, experimentally had no effect on SPM binding to the CTD, even at low experimental voltages; it was only at strong depolarizing conditions that SPM pore block was reduced in this mutant (Xie et al., 2002). In complementary simulations of the closely related channel Kir1.1 (ROMK), a weakly inward-rectifying channel that lacks the negatively charged, cavity-located aspartate residue D173, SPM binding to the open-pore conformation was almost exclusively observed at the CTD (Sims. 153–154; Fig. S4 B). This is likely due to the absence of two key negatively charged residues—equivalent to D173 and E225 in Kir2.2—along the permeation pathway of the extended pore, without which SPM cannot translocate from the CTD to the pore cavity. This leads to the low ROMK affinity to polyamines (*K*_i_ values are in the millimolar range), and weak SPM binding (and/or Mg^2+^ binding) can promote weak inward rectification in ROMK.

In our SPM-unbinding simulations (Sims. 60–90), potassium ions entered the SF from the extracellular side and displaced the SPM that had been bound to the pore cavity and SF (Fig. 6, A and B); permeation resumed upon inward SPM unbinding, indicating relief from SPM block. The presence of inward potassium currents underlies Kir2.2 inward rectification (Fig. 6 D). The voltage-dependent SPM-unbinding times observed in our simulations are broadly in line with experimental off-rates for SPM unbinding (Fig. 6 E; Shin et al., 2005), but the unbinding times are somewhat shorter (i.e., faster rates) in our simulations due to our application of artificially high hyperpolarizing voltages, which are required to observe unbinding on timescales accessible to simulation.

Were the main SPM-block site found to be below the pore cavity, faster unbinding times would be expected upon reduction of negative charge density in the CTD portion of the extended pore. In our simulations of the E225A/E300A double mutant with reduced negative CTD charge density (Sims. 93–120), however, the SPM unbinding times were slower than those in the WT simulations (e.g., at 215 mV, τoff = 26.2 µs for E225A/E300A, whereas τoff = 4.0 µs for the WT, consistent with the slower relief from SPM block observed experimentally (Fujiwara and Kubo, 2006; Kubo and Murata, 2001).

In further simulations of the E225A/R261Q double mutant, with a CTD charge density restored to be similar to that in the WT (Sims. 121–148), the SPM-unbinding times of τoff = 1.7 µs were indeed comparable to those we observed for the WT (Fig. 6 E and Fig. S5). This suggests that the CTD negative-charge density, along with being responsible for SPM sequestration and binding under depolarizing conditions, also modulates SPM-unbinding kinetics under hyperpolarizing conditions.

The details of SPM binding and unbinding observed in our simulations at depolarizing and hyperpolarizing voltages, respectively, suggest how inward rectification in Kir2.2 may occur physiologically in the presence of SPM. Our simulation results might also help to resolve the lack of consensus regarding the exact location of the polyamine (e.g., SPM) binding site in Kir channels (e.g., in Kir2.2; Guo and Lu, 2003; Kurata et al., 2013; Nichols and Lee, 2018; Baronas and Kurata, 2014). Our results appear to support the “deep” model (Baronas and Kurata, 2014), in which the binding site is close to the channel inner cavity—specifically, above the rectification controller D173, and extending halfway into the SF (Fig. 7).

### Conclusions

In this study, we used long-timescale MD simulations to study lipid-dependent channel activation and inward rectification due to the binding of a polyamine, SPM, in cKir2.2. We obtained a fully activated and ion-conducting channel conformation, agonized and stabilized by negatively charged PIP_2_ and POPS lipid molecules that, in our simulations, bound non-competitively to two distinct lipid-binding sites of the channel. Our simulation results also suggest that the N-terminally located IH and a C-terminally located R/K-rich region are critical for lipid-dependent Kir2.2 activation. Based on complementary observations of Kir2.2 deactivation in a PIP_2_-depleted environment, we have proposed a putative Kir2.2 activation mechanism (Fig. 7) that suggests indispensable functional roles for the primary and secondary lipid-binding sites.

Our simulations of SPM binding to a fully activated Kir2.2 conformation point to a sequential binding process that begins with charge-dependent recruitment of SPM to the CTD and ends with SPM binding to a deep binding site located between a rectification controller in the pore cavity, D173, and the SF. This binding process is compatible with experimental data on voltage-dependent SPM block (Fig. 7; Nichols and Lee, 2018; Baronas and Kurata, 2014).

## Supplementary Material

Table S1 lists molecular dynamics simulations

Table S2 lists spermine force field parameters

## Data Availability

The molecular dynamics trajectories described in this work are available for non-commercial use through contacting trajectories@deshawresearch.com.
