# Supplementary material for: Gating and modulation of an inward-rectifier potassium channel
Source: J Gen Physiol. 2022 Dec 16;155(2):e202213085. doi: 10.1085/jgp.202213085 (PMC9764021; doi:10.1085/jgp.202213085)
Supplement: Table S1 — lists molecular dynamics simulations [file JGP_202213085_TableS1.docx]

| **Sim #** | **Construct** | **Agonists/ligand** | ***t(***μs) | ***E*** | ***V*(mV)** | **Purpose** | ***k*_SF_** | ***No. of permeation events*** | ***I* (pA)** | **Starting structure** | **Observation** |
| --- | --- | --- | --- | --- | --- | --- | --- | --- | --- | --- | --- |
| 1 | WT | PIP2 | 3.2 | –0.05 | –310 | Control | 4 | - | - | X-ray | Pore closure |
| 2 | WT | PIP2, POPS | 199.2 | 0.05 | 310 | Control | 4 | - | - | X-ray | Pore closure |
| 3 | G178R | PIP2, POPS | 28.2 | 0.05 | 310 | Activation | 0 | - | - | X-ray | Pore opening |
| 4 | WT | PIP2, POPS | 140.1 | 0.05 | 310 | Permeation | 4 | 3036 | 4.1 ± 1.4 | 22.7 µs from Sim. 3 | Permeation |
| 5 | WT | PIP2, POPS | 153.8 | 0.05 | 310 | Permeation | 4 | 8308 | 8.65 ± 1.53 | 15 µs from Sim. 4 | Permeation |
| 6 | WT | PIP2, POPS | 120.0 | 0.06 | 375 | Permeation | 4 | 3895 | 5.22 ± 2.47 | 15 µs from Sim. 4 | Permeation |
| 7 | WT | PIP2, POPS | 48.0 | 0.05 | 310 | Permeation | 4 | 929 | 3.1 ± 1.0 | 15 µs from Sim. 4 | Permeation |
| 8 | WT | PIP2, POPS | 48.0 | 0.05 | 310 | Permeation | 4 | 1461 | 4.87 ± 0.61 | 15 µs from Sim. 4 | Permeation |
| 9 | WT | PIP2, POPS | 48.0 | 0.04 | 250 | Permeation | 4 | 984 | 3.28 ± 1.39 | 15 µs from Sim. 4 | Permeation |
| 10 | WT | PIP2, POPS | 48.0 | 0.035 | 215 | Permeation | 4 | 389 | 1.3 ± 0.44 | 15 µs from Sim. 4 | Permeation |
| 11 | WT | PIP2, POPS | 48.0 | 0.03 | 185 | Permeation | 4 | 163 | 0.65 ± 0.22 | 15 µs from Sim. 4 | Permeation |
| 12 | WT | PIP2, POPS | 36.0 | 0.025 | 155 | Permeation | 4 | 131 | 0.84 ± 0.52 | 15 µs from Sim. 4 | Permeation |
| 13 | WT | POPS | 90.0 | 0.05 | 310 | Deactivation | 4 | 1766 | 8.32 ± 3.98 | 15 µs from Sim. 4 | Pore closure |
| 14–19 | WT | POPS | 48.0–120.0 | 0.05 | 310 | Deactivation | 4 | 1521, 2777, 955, 2111, 1669, 434 | 3.35 ± 0.65 | 15 µs from Sim. 4 | Pore closure |
| 20–25 | D69A/D76A | POPS | 48.0–120.0 | 0.05 | 310 | Deactivation | 4 | 605, 103, 278, 703, 443, 3502 | 1.67 ± 0.62 | 15 µs from Sim. 4 | Pore opening |
| 26–27 | E225A/E300A | PIP2, POPS | 48.0 | 0.05 | 310 | Permeation | 4 | 177, 198 | 0.6 ± 0.11; 0.68 ± 0.15 | 15 µs from Sim. 4 | Permeation |
| 28–29 | E225A/R261Q | PIP2, POPS | 48.0 | 0.05 | 310 | Permeation | 4 | 1155, 1460 | 3.85 ± 1.13; 4.87 ± 0.82 | 15 µs from Sim. 4 | Permeation |
| 30 | WT | PIP2, POPS / SPM | 10.0 | 0.05 | 310 | SPM binding | 4 | - | - | 15 µs from Sim. 4 | Pore block |
| 31–34 | WT | PIP2, POPS / SPM | 48.0–24.0 | 0.05 | 310 | SPM binding | 4 | - | - | 15 µs from Sim. 4 | Pore block |
| 35–38 | WT | PIP2, POPS / SPM | 24.0 | 0.04 | 250 | SPM binding | 4 | - | - | 15 µs from Sim. 4 | Pore block |
| 39–42 | WT | PIP2, POPS / SPM | 24.0 | 0.035 | 215 | SPM binding | 4 | - | - | 15 µs from Sim. 4 | Pore block |
| 43–46 | WT | PIP2, POPS / SPM | 24.0 | 0.03 | 185 | SPM binding | 4 | - | - | 15 µs from Sim. 4 | Pore block |
| 47-–50 | WT | PIP2, POPS / SPM | 24.0 | 0.025 | 155 | SPM binding | 4 | - | - | 15 µs from Sim. 4 | No SPM binding |
| 51–52 | WT | PIP2, POPS / SPM | 24.0 | –0.05 | –310 | SPM binding | 4 | 1737, 493 | −7.44 ± 4.15 | 15 µs from Sim. 4 | No SPM binding |
| 53–54 | WT | PIP2, POPS / SPM | 24.0 | –0.04 | –250 | SPM binding | 4 | 294, 510 | −2.68 ± 0.72 | 15 µs from Sim. 4 | No SPM binding |
| 55–56 | WT | PIP2, POPS / SPM | 24.0 | –0.035 | –215 | SPM binding | 4 | 266, 317 | −1.96 ± 0.15 | 15 µs from Sim. 4 | No SPM binding |
| 57–58 | WT | PIP2, POPS / SPM | 24.0 | –0.03 | –185 | SPM binding | 4 | 292, 222 | −1.71 ± 0.24 | 15 µs from Sim. 4 | No SPM binding |
| 59 | WT | PIP2, POPS / SPM | 24.0 | –0.025 | –155 | SPM binding | 4 | 80 | −0.54 ± 0.15 | 15 µs from Sim. 4 | No SPM binding |
| 60 | WT | PIP2, POPS / SPM | 3.4 | –0.05 | –310 | SPM unbinding | 4 | 48 | −3.01 ± 1.31 | 6 µs from Sim. 30 | Relief from pore block |
| 61–67 | WT | PIP2, POPS / SPM | 1.0–8.0 | –0.05 | –310 | SPM unbinding | 4 | 28, 21, 20, 63, 31, 52, 19 | −8.73 ± 6.06 | 6 µs from Sim. 30 | Relief from pore block |
| 68–74 | WT | PIP2, POPS / SPM | 1.0–11.0 | –0.035 | –215 | SPM unbinding | 4 | 49, 23, 4, 8, 15, 153, 38 | −2.16 ± 1.09 | 6 µs from Sim. 30 | Relief from pore block |
| 75–81 | WT | PIP2, POPS / SPM | 3.0–48.0 | –0.03 | –185 | SPM unbinding | 4 | 18, 18, 35, 0, 40, 140, 9 | −1.87 ± 1.62 | 6 µs from Sim. 30 | Relief from pore block |
| 82 | WT | PIP2, POPS / SPM | 6.4 | –0.025 | –155 | SPM unbinding | 4 | 14 | −0.52 ± 0.17 | 6 µs from Sim. 30 | Relief from pore block |
| 83–90 | WT | PIP2, POPS / SPM | 2.0–48.0 | –0.025 | –155 | SPM unbinding | 4 | 14, 1, 0, 0, 0, 30, 158, 7 | −1.24 ± 0.73 | 6 µs from Sim. 30 | Relief from pore block |
| 91–92 | E225A/E300A | PIP2, POPS / SPM | 48.0 | 0.05 | 310 | SPM binding | 4 | 75, 72 | 0.25 ± 0.05; 0.25 ± 0.12 | 15 µs from Sim. 4 with E225A/E300A mutation | No SPM binding |
| 93–99 | E225A/E300A | PIP2, POPS / SPM | 1.0–24.0 | −0.05 | −310 | SPM unbinding | 4 | 116, 85, 177, 47, 35, 30, 37 | −4.44 ± 1.93 | 6 µs from Sim. 30 with E225A/E300A mutation | Relief from pore block |
| 100–106 | E225A/E300A | PIP2, POPS / SPM | 5.0–48.0 | −0.035 | −215 | SPM unbinding | 4 | 6, 149, 9, 14, 39, 3, 44 | −1.42 ± 0.91 | 6 µs from Sim. 30 with E225A/E300A mutation | Relief from pore block |
| 107–113 | E225A/E300A | PIP2, POPS / SPM | 12.0–48.0 | −0.03 | −185 | SPM unbinding | 4 | 0, 4, 14, 4, 4, 8, 101 | −0.67 ± 0.23 | 6 µs from Sim. 30 with E225A/E300A mutation | Relief from pore block |
| 114–120 | E225A/E300A | PIP2, POPS / SPM | 48.0 | −0.025 | −155 | SPM unbinding | 4 | - | - | 6 µs from Sim. 30 with E225A/E300A mutation | No relief from pore block |
| 121–127 | E225A/R261Q | PIP2, POPS / SPM | 1.0–6.0 | −0.05 | −310 | SPM unbinding | 4 | 123, 34, 116, 7, 101, 50, 39 | −4.75 ± 1.56 | 6 µs from Sim. 30 with E225A/R261Q mutation | Relief from pore block |
| 128–134 | E225A/R261Q | PIP2, POPS / SPM | 1.0–20.0 | −0.035 | −215 | SPM unbinding | 4 | 5, 16, 21, 53, 69, 7, 25 | −2.63 ± 2.25 | 6 µs from Sim. 30 with E225A/R261Q mutation | Relief from pore block |
| 135–141 | E225A/R261Q | PIP2, POPS / SPM | 1.0–48.0 | −0.03 | −185 | SPM unbinding | 4 | 5, 19, 7, 4, 31, 8, 40 | −1.02 ± 0.63 | 6 µs from Sim. 30 with E225A/R261Q mutation | Relief from pore block |
| 142–148 | E225A/R261Q | PIP2, POPS / SPM | 3.0–48.0 | −0.025 | −155 | SPM unbinding | 4 | 5, 18, 12, 72, 5, 2, 1 | −0.40 ± 0.34 | 6 µs from Sim. 30 with E225A/R261Q mutation | Relief from pore block |
| 149 | WT, D173 deprot. | PIP2, POPS | 60.0 | 0.05 | 310 | Permeation | 4 | 943 | 2.14 ± 0.44 | 15 µs from Sim. 4 | Permeation |
| 150 | WT, D173 deprot. | PIP2, POPS / SPM | 48.0 | 0.05 | 310 | SPM binding | 4 | - | - | 15 µs from Sim. 4 | Pore block |
| 151 | WT, D173 deprot. | PIP2, POPS / SPM | 36.0 | 0.04 | 250 | SPM binding | 4 | - | - | 15 µs from Sim. 4 | Pore block |
| 152 | WT, D173 deprot. | PIP2, POPS / SPM | 24.0 | 0.035 | 215 | SPM binding | 4 | 126 | 0.89 ± 0.43 | 15 µs from Sim. 4 | No SPM binding |
| 153 | ROMK | PIP2, POPS / SPM | 48.0 | 0.05 | 310 | SPM binding | 4 | - | - | Modeled on open cKir2.2 | No SPM binding |
| 154 | ROMK | PIP2, POPS / SPM | 48.0 | 0.05 | 310 | SPM binding | 4 | - | - | Modeled on open cKir2.2 | No SPM binding |

**Table S1. Molecular dynamics simulations.** Total simulation time (*t*, μs); applied field (*E*, kcal × mol^-1^ × Å^-1^ × e^-1^); applied voltage (*V* = *E* × average simulation box length in the z direction, mV); ionic (K^+^) current (*I*, pA; in the SPM-unbinding simulations, *I* was calculated as the average current after SPM had unbound, and in simulations with no PIP_2_, *I* was calculated before closure); force constant for correction of the torsional backbone potential in the selectivity filter (*k*_SF_; kcal mol^−1^) applied to residues 143–147. In Sims. 1, 2, 4, 13, 30, and 60, torsional φ SF corrections were applied to residues G145 and G147 only. In Sim. 3, backbone position restraints were applied to all SF residues with a force constant of 1 kcal × mol^-1^ × Å^-2^. In Sim. 5, a 0.5 M KCl bulk ionic concentration was used; in all other simulations this concentration was kept at 0.2 M KCl. A ROMK model based on the activated Kir2.2 conformation (obtained in Sim. 3) was built using MOE (Vilar et al., 2008), and SPM binding to the open pore of this ROMK model was simulated in Sims. 153 and 154. To preserve SF and CTD structural integrity, φ torsional backbone corrections were applied to SF residues G143 and G145, whereas C_α_ RMSD restraints of 60 kcal × mol^-1^ × Å^-2^ were imposed on each ROMK CTD subunit independently. The “No. of permeation events” column reflects the number of potassium ions that permeated during the portions of the simulations in which the pore remained open in the SPM-free simulations, or remained open and unblocked in the simulations with SPM.

Analysis of MD trajectories: (i) the average waiting time between two successive permeation events was used to determine the K^+^ current at each voltage, and the standard error was estimated by block analysis of the waiting times between such events (Flyvbjerg and Peterson, 1989); (ii) ion occupancy was measured as the average number of ions observed (over the total simulation time of a given simulation) within a particular region of interest: SF, T143-Oγ1 to G147-O, pore cavity, S174-Cα to T143-Oγ1, CTD, D256-Cα to A182-Cα; (iii) to estimate SPM-binding kinetics, we defined an SPM-binding event as when the distance from the approaching SPM molecule to the D173 center of mass (C_α_ atom-based) was 10 Å or less, using 30‑ns running medians. All analyses were performed using in-house software.

Flyvbjerg, H., and H.G. Petersen. 1989. Error estimates on averages of correlated data. *J. Chem. Phys*. 91:461–466. 10.1063/1.457480
