# Supplementary material for: Gating and modulation of an inward-rectifier potassium channel
Source: J Gen Physiol. 2022 Dec 16;155(2):e202213085. doi: 10.1085/jgp.202213085 (PMC9764021; doi:10.1085/jgp.202213085)
Supplement: Table S2 — lists spermine force field parameters [file JGP_202213085_TableS2.docx]

**Table S2. Spermine force field parameters:**

**Charge and atom types:**

| **Atom name** | **Atomic number** | **Charge-(e)** | **Atom type** |
| --- | --- | --- | --- |
| N1 | 7 | −0.304 | NG3P3 |
| C1 | 6 | 0.116 | CG324 |
| C2 | 6 | −0.202 | CG321 |
| C3 | 6 | 0.228 | CG324 |
| N2 | 7 | −0.402 | NG3P2 |
| C4 | 6 | 0.200 | CG324 |
| C5 | 6 | −0.189 | CG321 |
| C6 | 6 | −0.189 | CG321 |
| C7 | 6 | 0.200 | CG324 |
| N3 | 7 | −0.402 | NG3P2 |
| C8 | 6 | 0.228 | CG324 |
| C9 | 6 | −0.202 | CG321 |
| C10 | 6 | −0.116 | CG324 |
| N4 | 7 | −0.304 | NG3P3 |
| H1 | 1 | 0.090 | HGA2 |
| H2 | 1 | 0.090 | HGA2 |
| H3 | 1 | 0.090 | HGA2 |
| H4 | 1 | 0.090 | HGA2 |
| H5 | 1 | 0.090 | HGA2 |
| H6 | 1 | 0.090 | HGA2 |
| H7 | 1 | 0.090 | HGA2 |
| H8 | 1 | 0.090 | HGA2 |
| H9 | 1 | 0.090 | HGA2 |
| H10 | 1 | 0.090 | HGA2 |
| H11 | 1 | 0.090 | HGA2 |
| H12 | 1 | 0.090 | HGA2 |
| H13 | 1 | 0.090 | HGA2 |
| H14 | 1 | 0.090 | HGA2 |
| H15 | 1 | 0.090 | HGA2 |
| H16 | 1 | 0.090 | HGA2 |
| H17 | 1 | 0.090 | HGA2 |
| H18 | 1 | 0.090 | HGA2 |
| H19 | 1 | 0.090 | HGA2 |
| H20 | 1 | 0.090 | HGA2 |
| H21 | 1 | 0.330 | HGP2 |
| H22 | 1 | 0.330 | HGP2 |
| H23 | 1 | 0.330 | HGP2 |
| H24 | 1 | 0.330 | HGP2 |
| H25 | 1 | 0.331 | HGP2 |
| H26 | 1 | 0.331 | HGP2 |
| H27 | 1 | 0.331 | HGP2 |
| H28 | 1 | 0.331 | HGP2 |
| H29 | 1 | 0.331 | HGP2 |
| H30 | 1 | 0.331 | HGP2 |

**Bonds (equilibrium lengths) in Å, force constant (fc) in kcal × mol^-1^ × Å^-2^:**

[“NG3P2”, “CG324”], “params”: {“r0”: 1.49, “fc”: 200.0}

[“NG3P3”, “CG324”], “params”: {“r0”: 1.49, “fc”: 200.0}

[“CG321”, “CG321”], “params”: {“r0”: 1.53, “fc”: 222.5}

[“CG324”, “CG321”], “params”: {“r0”: 1.53, “fc”: 222.5}

[“CG324”, “HGA2”], “params”: {“r0”: 1.1, “fc”: 284.5}

[“CG321”, “HGA2”], “params”: {“r0”: 1.111, “fc”: 309.0}

[“NG3P2”, “HGP2”], “params”: {“r0”: 1.006, “fc”: 460.0}

[“NG3P3”, “HGP2”], “params”: {“r0”: 1.04, “fc”: 403.0}

**Angles (equilibrium angle) in deg., force constant (fc) in kcal × mol^-1^ × rad^-2^:**

[“CG324”, “CG321”, “CG324”], “params”: {“theta0”: 110.50, “fc”: 58.35}

**Dihedrals (equilibrium angle) in deg., force constants (fc) in kcal × mol^-1^:**

[“CG324”, “CG321”, “CG321”, “CG324”], “params”: {“phi0”: 0.0, “fc0”: 0.1950, “fc1”: 0.0, “fc2”: 0.0, “fc3”: 0.1950, “fc4”: 0.0, “fc5”: 0.0, “fc6”: 0.0}

[“CG324”, “CG321”, “CG324”, “NG3P2”], “params”: {“phi0”: 0.0, “fc0”: 1.0000, “fc1”: 0.0, “fc2”: 0.0, “fc3”: 1.0000, “fc4”: 0.0, “fc5”: 0.0, “fc6”: 0.0}

[“CG324”, “CG321”, “CG324”, “NG3P3”], “params”: {“phi0”: 0.0, “fc0”: 0.1950, “fc1”: 0.0, “fc2”: 0.0, “fc3”: 0.1950, “fc4”: 0.0, “fc5”: 0.0, “fc6”: 0.0}

[“CG324”, “CG321”, “CG324”, “HGA2”], “params”: {“phi0”: 0.0, “fc0”: 0.1950, “fc1”: 0.0, “fc2”: 0.0, “fc3”: 0.1950, “fc4”: 0.0, “fc5”: 0.0, “fc6”: 0.0}
